# High-Temperature, Solid-Phase Reaction of α-Amino Groups in Peptides with Lactose and Glucose: An Alternative Mechanism Leading to an α-Ketoacyl Derivative

**DOI:** 10.1021/acs.jafc.3c00821

**Published:** 2023-03-31

**Authors:** Monika Kijewska, Michalina Zawadzka, Piotr Stefanowicz

**Affiliations:** Faculty of Chemistry, University of Wrocław, Joliot-Curie 14, 50-383 Wrocław, Poland

**Keywords:** LC−MS/MS analysis, lactosylated peptides, α-ketoacid derivatives, deamination catalyzed by a reducing sugar

## Abstract

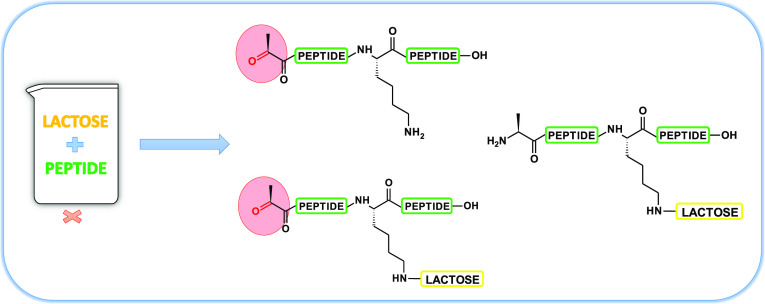

The reaction of proteins with reducing sugars results in the formation of Amadori products, which involves the N-terminal group and/or ε-amino group of the lysine side chain. However, less attention has been given to the reactivity of the N-terminus of a peptide chain under similar conditions. In our work, we focused on the reaction of the α-amino group of peptides in the presence of a reducing sugar, specifically lactose. We optimized the reaction conditions of model peptides with lactose in the solid phase and showed that temperatures above 120 °C lead to the deamination of the N-terminal amino acid moiety, ultimately resulting in α-ketoacids. We carried out detailed studies to confirm the structure of the deaminated product using analytical methods such as ESI-MS and LC–MS/MS, as well as chemical methods that involved the reduction of the carbonyl group combined with isotopic exchange and the reactivity of the carbonyl group with the hydroxylamine derivative. The structure of the reaction product was also confirmed by chemical synthesis. We suggested plausible mechanisms for the formation of the deaminated product and considered the probable path of its formation. Our aim was to determine whether the reaction proceeds according to the Strecker-based mechanism and direct imine isomerization by carrying out reactions of model peptides in the presence of lactose under aerobic and anaerobic conditions and comparing the results obtained.

## Highlights

- LC–MS/MS analysis of lactosylated peptides.
- Deamination of the N-terminal amino acid by a reducing sugar.
- Chemoselective reaction of the hydroxylamine derivative with the deamination product.

## 1 Introduction

Milk and milk products are rich sources of nutrients that play an important role in human diets. Ultrahigh-temperature treatment (UHT) or pasteurization provides microbiological safety for these products; thus, this step is crucial in milk product processing. Infant formula undergoes even harsher conditions. Nevertheless, high temperature and the presence of a reducing sugar—lactose—promote non-enzymatic reactions, such as oxidation and Maillard reaction.^1−5^ Due to the reaction between the carbonyl group of lactose and a free amino group of Lys residues, a stable Amadori product—ε-lactulosyllysine—is formed. As a result, lactosylated proteins lose their nutritional values, bioavailability, digestibility, and overall product quality. Moreover, modification of proteins such as β-lactoglobulin, α-lactalbumin, and caseins, the main abundant milk proteins, affects their allergenicity, which is of great importance in the nutrition of infants.

The products formed in the reaction of reducing sugars and proteins can be detected by various analytical techniques. Commercially available boronate-affinity materials (BAMs) are used to enrich samples in lactosylated peptides,^6,7^ which allows the detection of low-concentration components of samples containing sugar moieties. Recently, Kijewska and co-workers have developed a functionalized resin—PhB-Lys(PhB)-ChemMatrix Rink resin—that selectively and efficiently captures glycated peptides.^8,9^ Furthermore, tags containing quaternary ammonium salt and phenylboronic acid derivatives, appropriately designed and synthesized, were used to increase the ionization efficiency of glycoconjugates in mass spectrometry analysis.^10,11^ Furosine, formed by the hydrolysis of Amadori compounds, is usually detected by high-pressure liquid chromatography (HPLC),^12^ ultra-pressure liquid chromatography (UPLC), or gas chromatography (GC) combined with fluorescence detection or mass spectrometry (MS).^7,13^ Lactosylation sites can be analyzed directly by MS^14^ or MS coupled with separation techniques, such as liquid chromatography (LC),^2,4,6,15,16^ two-dimensional (2D) gel electrophoresis,^17,18^ or capillary electrophoresis (CE).^3,19^ The immunoassay techniques^12,19^ are also applied. The advantage of mass spectrometry is its high sensitivity and specificity, which allows the identification and quantification of protein modification products. MS methods allow identifying lactosylated peptides based on characteristic +324 Da mass shift (mass of ε-lactosyllysine and ε-lactulosyllysine) compared to nonmodified peptides.^20^ Tandem mass spectrometry provides information about the exact lactosylation site and amino acid residue, which is modified since oligosaccharides have specific fragmentation pathways that can be used for their identification and differentiation.^21^ The characteristic neutral loss of −216 Da, identified as the furylium ion, indicates the presence of lactosylated peptides.^22^

In our study, we optimized the reaction conditions of model peptides in the presence of lactose, resulting in the oxidative deamination of the N-terminus combined with carbonyl group formation in this position. So far in the literature,^23,24^ this reaction was observed only in solution; therefore, our study was focused on the deamination reaction that undergoes in the solid phase. Even though the deamination of the N-terminal amino acid moiety was already noticed by Meltretter et al.,^23,24^ those experiments were performed in a very complex matrix (milk), hampering the demonstration of the direct impact of the reducing sugar on the deamination reaction taking place at the N-terminus of the peptide. Moreover, deamination products have been observed in trace amounts, in addition to numerous advanced glycation products, and have not been confirmed by other research methods. The research on model peptides presented here for the first time confirms our hypothesis that the reducing sugar has an impact on the deamination reaction. Moreover, we proved that this carbonyl product can be formed also in the presence of other reducing sugars like glucose depending on reaction conditions. The presence of the α-ketocarboxylic amide derivative was confirmed by LC–MS/MS analysis and ^18^O isotopic exchange combined with reduction. We also demonstrated a new mechanism leading to the formation of a peptide containing an α-ketoacyl derivative.

## 2 Materials and Methods

### 2.1 General Information

#### 2.1.1 LC–MS Analysis

All LC–MS and LC–MS/MS experiments were performed on a Shimadzu IT-TOF or Shimadzu qTOF-9030 instrument with an electrospray ion source in positive ion mode. Separation was carried out on an RP-Zorbax (50 × 2.1 mm, 3.5 μm) column with a gradient elution of 0–40% B in A or 0–60% B in A (A = 0.1% HCOOH in water; B = 0.1% HCOOH in MeCN) at room temperature for 20 or 15 min (flow rate: 0.1 mL/min).

### 2.2 Experimental Part

#### 2.2.1 Reagents

The derivatives of amino acids for peptide synthesis and the coupling reagent (TBTU, *O*-(benzotriazol-1-yl)-*N*,*N*,*N*′,*N*′-tetramethyluronium tetrafluoroborate) were purchased from NovaBiochem. Lactose and glucose were purchased from Sigma-Aldrich. The ChemMatrix Rink Resin (0.4–0.60 mmol/g) was purchased from Sigma-Aldrich. The solvents for peptide synthesis (analytical grade) were obtained from Riedel de Haen (DMF) and J. T. Baker (methanol and acetonitrile). LC–MS solvents (water, acetonitrile, and methanol) were purchased from ChemSolve and J.T. Baker. Other reagents used in this work were obtained from Aldrich (triisopropylsilane (TIS)) and IrisBiotech (trifluoroacetic acid and *N*,*N*-diisopropylethylamine (DIEA)).

#### 2.2.2 Synthesis of Peptides

Peptides were synthesized manually on the solid support (Wang Resin or ChemMatrix Rink Resin) according to Fmoc protocol ultrasonic agitation developed by Wołczański et al.^25^ using DIEA (6 equiv) and TBTU (3 equiv) as coupling reagents. After SPPS synthesis, acetylation of the N-terminus was performed using the mixture of Ac_2_O:DIEA:DMF (0.9:1.7:7.4, v:v:v). After acetylation, the resin was washed with DMF, DCM, THF, and Et_2_O and dried. Peptides were cleaved from resin with a TFA:H_2_O:TIS (95:2.5:2.5) mixture. The progress of the reaction was controlled by the Kaiser test. Obtained peptides were analyzed by LC–MS/MS.

#### 2.2.3 Synthesis of Lactosylation Peptides (High-Temperature Reaction)

Peptide and lactose were mixed in a 1:10 molar ratio. The mixture was dissolved in 1 mL of water and then lyophilized. The lyophilized sample was placed in an oven in the following reaction conditions: 80 °C for 20 min, 100 °C for 1, 4, and 12 h, and 120 °C for 1 and 4 h. After that, the reaction samples were subjected to ESI-MS and LC–MS/MS analyses. All experiments were performed three times and analyzed twice to confirm obtained results.

#### 2.2.4 Synthesis of the Model Pyr-KAF-NH_2_

Peptides were synthesized manually on ChemMatrix Rink Resin according to Fmoc protocol ultrasonic agitation developed by Wołczański et al.^25^ using DIEA (6 equiv) and TBTU (3 equiv) as coupling reagents. After attaching Fmoc-Lys(Boc)-OH, the Fmoc protecting group was removed using 25% piperidine in DMF for 3 min in an ultrasonic bath and pyruvic acid, after previous activation using TBTU (6 equiv) and DIEA (12 equiv), was attached in an ultrasonic bath for 30 min. After that, the peptidyl resin was washed with DMF, DCM, THF, and Et_2_O and dried. Peptides were cleaved from resin with a TFA:H_2_O:TIS (95:2.5:2.5) mixture. The obtained peptide was analyzed by LC–MS/MS.

#### 2.2.5 General Capturing Procedure of AOA-Linker-CMRR with a Compound Containing a Carbonyl Group (Based on the Procedure Published by Kijewska et al.^26^)

Five milligrams of Fmoc-AOA-GRG-CMRR was swelled in a syringe (reaction columns, Intravis, Bioanalytical Instruments) for 30 min in DMF (2 mL). After deprotection of the N-terminal amino group using 2 mL of 25% piperidine in DMF for 3 min in an ultrasonic bath, the functionalized resin was washed with 1 mL of DMF (7 × 1 min) and 1 mL of AcOH (3 × 1 min). The mixture of products after reaction with lactose was dissolved in 1 mL of acetic acid, added to the functionalized resin, and mixed for 4 h. Then, the solution was filtered off (uncaptured fraction), while the resin was washed using 1 mL of the following set of solvents: AcOH (3 × 1 min), DCM (3 × 1 min), THF (3 × 1 min), and Et_2_O (3 × 1 min). The peptidyl-resin was dried in a vacuum desiccator for 1 day at room temperature. The products were cleaved from the resin using 2 mL of water/trifluoroacetic acid (5:95) for 2 h. The solution was evaporated under a gentle stream of nitrogen. Finally, the products were lyophilized and subjected to LC–MS/MS analysis.

## 3 Results and Discussion

Solid-phase glycation of peptides and proteins described by Boratyński and Roy^27^ was tested on ubiquitin,^28^ lysozyme, and many peptides,^29^ giving surprisingly homogeneous Amadori products, free from oxidation and dehydration. A similar reaction was carried out on a series of peptides (H-AKAF-OH, Ac-AKAF-OH, H-AKAF-NH_2_, H-AAFR-OH, H-LVTDLTK-OH, H-RAKAFKA-NH_2_, and H-SEVLRLVKDPAK-OH), synthesized on the solid support according to Wołczański’s method,^25^ using lactose; however, the results of the experiment were different from that observed for glucose–peptide systems. The appropriate sequence of peptides was selected for the following reasons: (i) demonstration of the reactivity of the α-amino group of the peptide, ε-amino group of the lysine residue, and guanidine group of arginine; therefore, the models include a compound in which the lysine residue has been replaced with an arginine residue, as well as models containing both amino acid residues; (ii) the introduction of an acyl residue at the N-terminus preventing deamination reactions; (iii) demonstration that regardless of the length of the peptide or various amino acid residues located at the N-terminus, the deamination reaction is observed; (iv) model conditions imitating hydrolysates of infant formula.^30−32^

**Figure 1:**
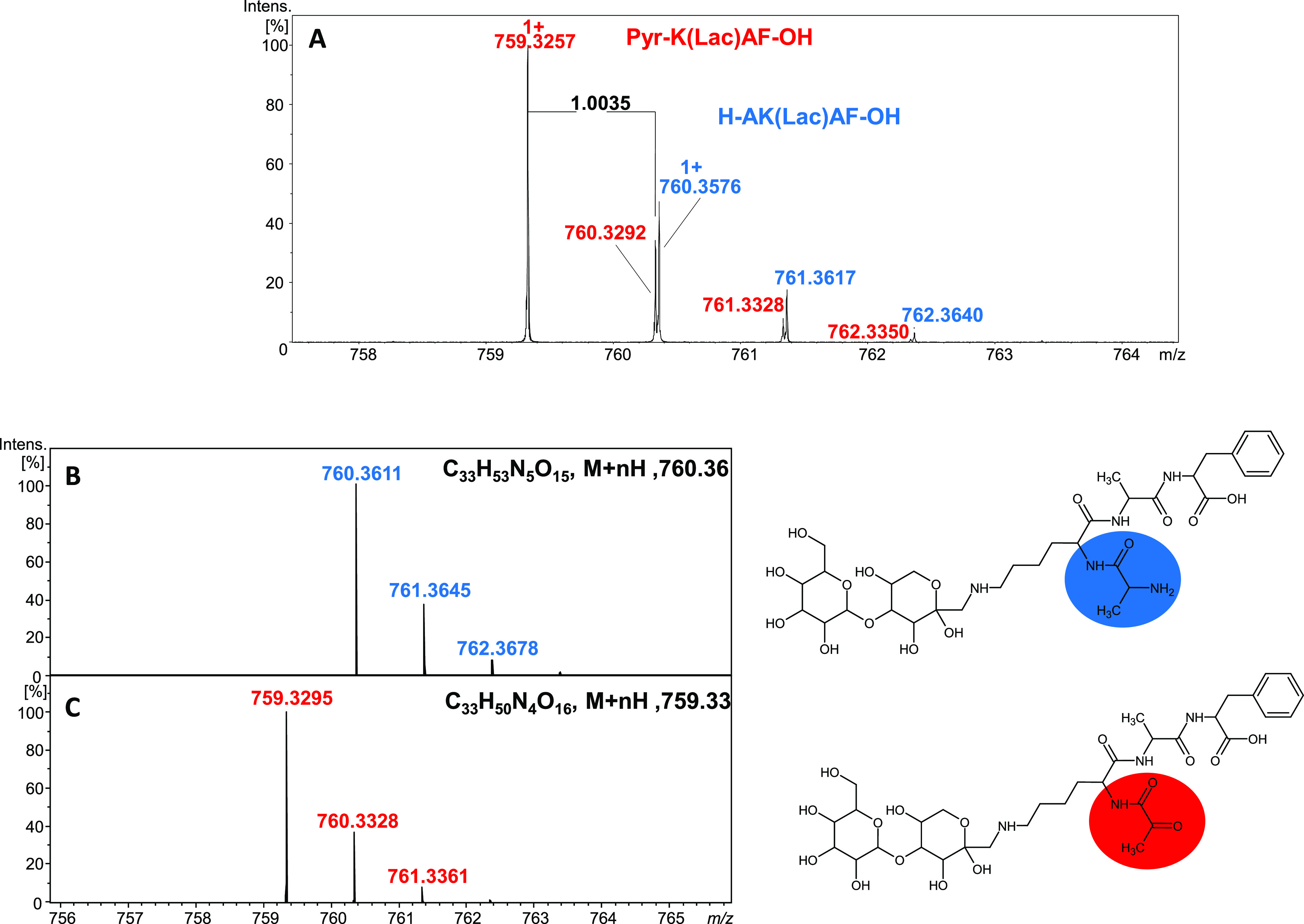
Expanded range of the ESI-MS spectrum of the mixture of lactosylated peptide and deaminated lactosylated peptide (A); simulated isotopic pattern for molecular formulas (B, C) and the structural formula of investigated compounds.

The Lys moieties underwent a well-described reaction, forming an Amadori product, while the reaction on the N-terminal α-amino group resulted in the formation of a product with the molecular mass shifted by 1.0035 Da (Figure 1). This mass difference was interpreted as a replacement NH_2_ group by the keto group (oxidative deamination), which is in good agreement with simulated isotopic patterns for investigated compounds—monolactosylated at the ε-amino group of the lysine moiety in H-AK(*Lac*)AF-OH and the second one containing a residue pyruvic acid derivative (Pyr-K(*Lac*)AF-OH) instead of alanine.

**Figure 2:**
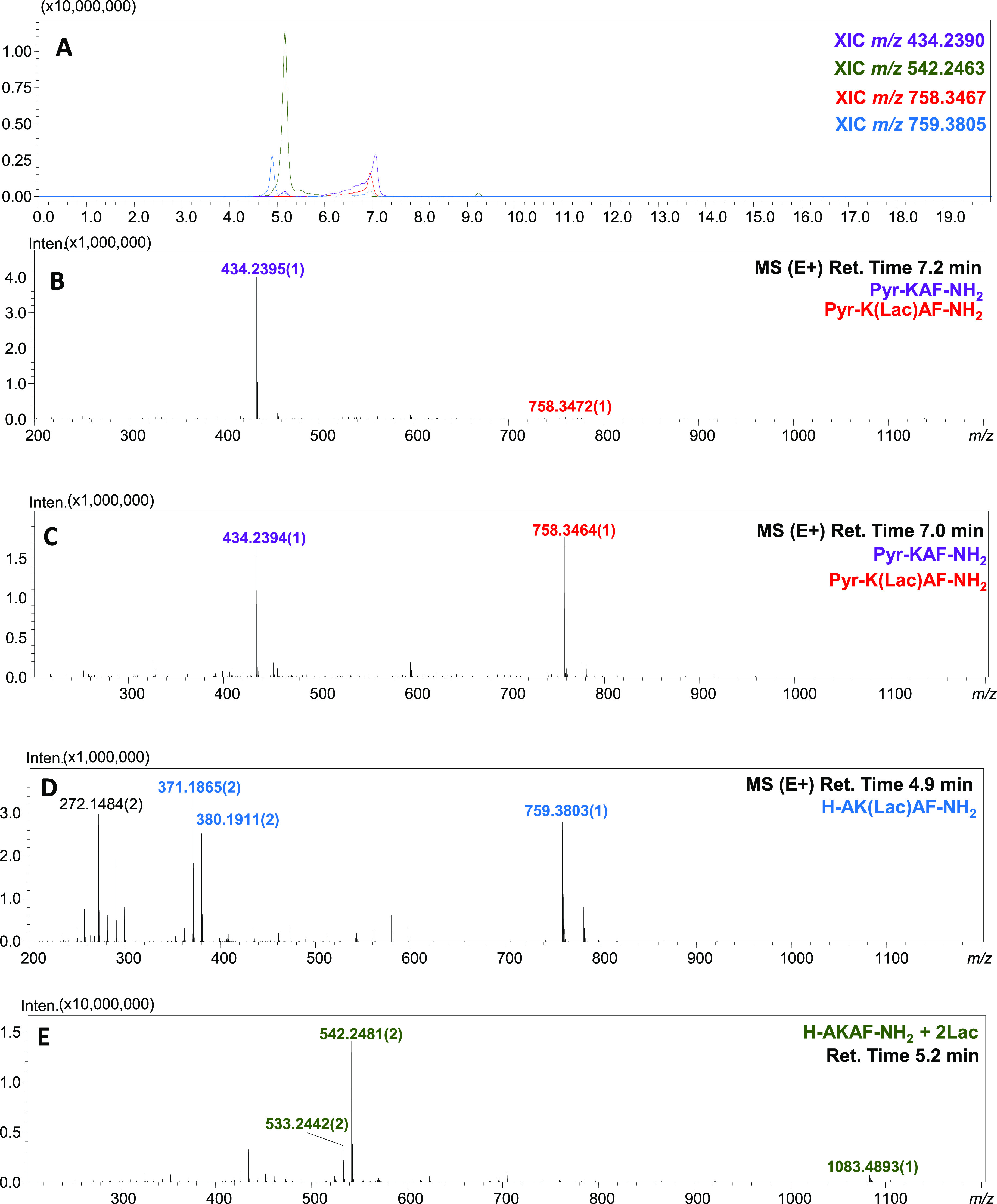
LC–MS chromatogram for the crude reaction mixture after the reaction of H-AKAF-NH_2_ with lactose (conditions: 120 °C for 4 h) (A); ESI-MS spectra (B–E).

We optimized the temperature and time of reaction showing that the deamination at the N-terminus depends on both parameters and becomes predominant after incubation of the sample for 4 h at 120 °C. For this purpose, the model peptides (A-AKAF-OH, Ac-AKAF-OH, H-LVTDLTK-OH, and H-AKAF-NH_2_) were mixed with lactose in the 1:10 molar ratio and then placed in the oven in the following reaction conditions: 80 °C for 20 min, 100 °C for 1, 4, and 12 h, and 120 °C for 1 and 4 h. The exemplary obtained results for model peptides containing both carboxylic acid and amide at the C-terminus in different reaction conditions are given in the Supporting Information (Figures S1–S7). The LC–MS chromatogram of the model peptide H-AKAF-NH_2_ after reaction with lactose is presented in Figure 2. Besides the signals corresponding to monolactosylated (*m*/*z* 759.3803) and dilactosylated peptides (*m*/*z* 542.2481, charge 2+) (Figure 1B,E), we observed the deamination products for both nonlactosylated (*m*/*z* 434.2395) and lactosylated peptides (*m*/*z* 758.3464) (Figure 1B,C). Because of the similar physicochemical properties of nonmodified and monolactosylated products, they co-elute, so separation using HPLC chromatography is limited. The same observation was made for glycated peptides. Therefore, in our recent paper,^8^ we developed the method of purification of the glycated peptide using boronate affinity chromatography on our functionalized resin.

**Figure 3:**
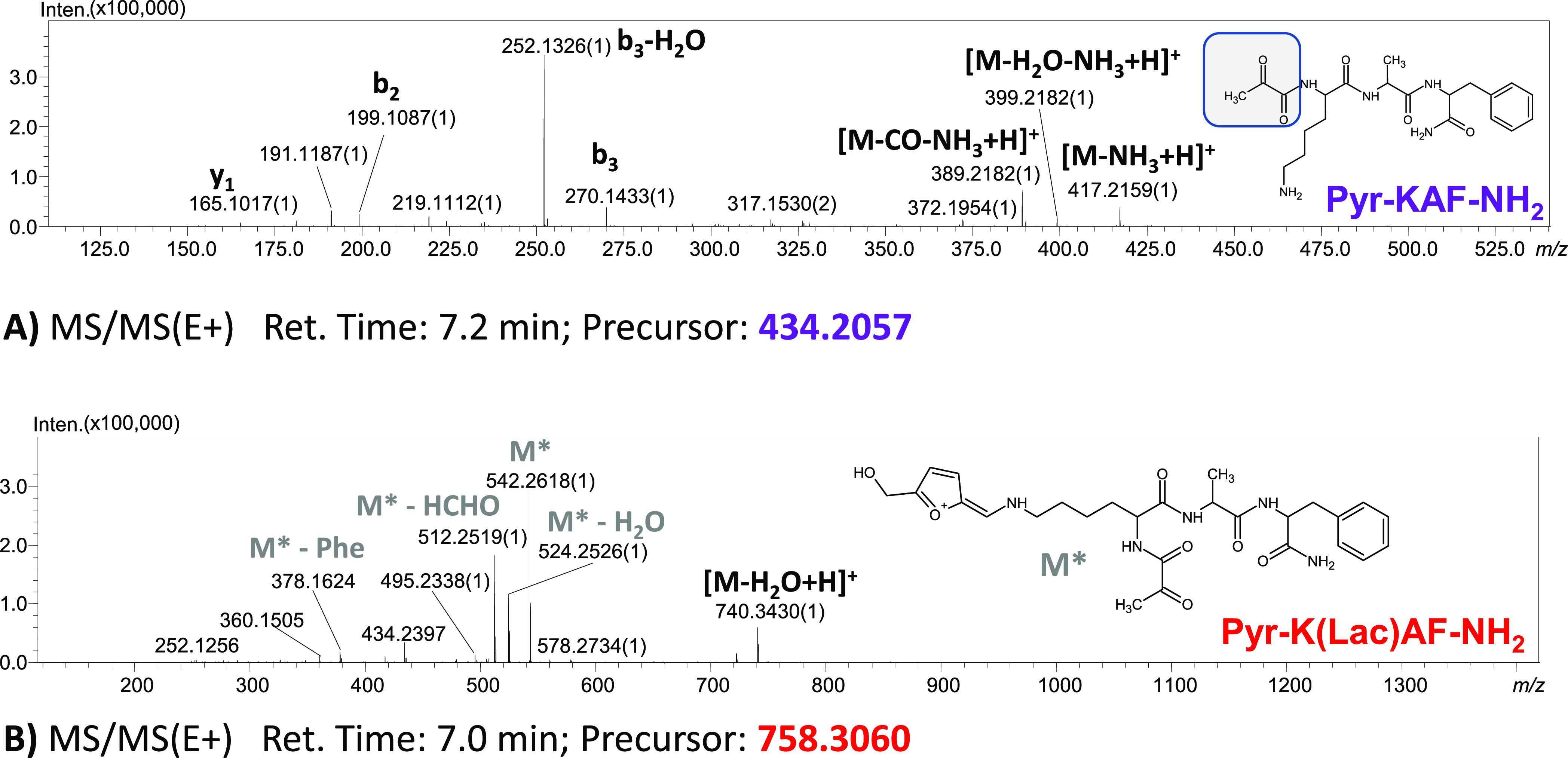
ESI-MS/MS spectra for deamination products: Pyr-KAF-NH_2_ (A) and Pyr-K(*Lac*)AF-NH_2_ (B).

The obtained deamination products (Pyr-KAF-NH_2_ and Pyr-K(*Lac*)AF-NH_2_) were subjected to LC–MS/MS analysis, revealing that the chemical modification concerns N-terminal amino acid. The CID spectra of the representative samples are presented in Figure 3. The MS/MS spectra are consistent with our assumption. The fragmentation pattern indicates that the deaminated moiety is located at the N-terminus of the peptide. Moreover, the lysine side chain-modified peptide is also deaminated at the N-terminus. The presence of the lactosyllysine moiety is confirmed by the characteristic furylium ion (*m*/*z* 542.2618) formed during tandem MS experiments. Therefore, the MS and MS/MS spectra give strong support for the proposed structure containing the N-terminal α-ketoacyl moiety.

Additional independent confirmation of the identity of the peptide containing α-ketoacyl at the N-terminus was the synthesis of a model peptide (Pyr-KAF-NH_2_) containing pyruvic acid according to the Fmoc strategy and comparison of the retention time and fragmentation spectrum with the data obtained for the compound after incubation of H-AKAF-NH_2_ with lactose. The obtained results presented in Figures S8–S10 clearly show that these two compounds are identical, i.e., deamination occurs at the N-terminus.

In our further investigation, two more peptides (H-RAKAFKA-NH_2_ and H-SEVLRLVKDPAK-OH) were tested using the conditions obtained during optimization (4 h at 120 °C) to show that deamination can occur at different amino acid residues. The obtained results were placed in Figures S11–S13. In both cases, we observe a mixture of products containing unmodified peptides and their analogs containing one, two, or three lactose molecules as well as appropriate deaminated analogs, which were confirmed by LC–MS/MS analysis, isotopic distributions, and the observed co-elution. The performed analysis showed deamination leading to 2-oxoarginine and 2-hydroxypyruvic acid for arginine and serine residues located at the N-terminus, respectively.

**Figure 4:**
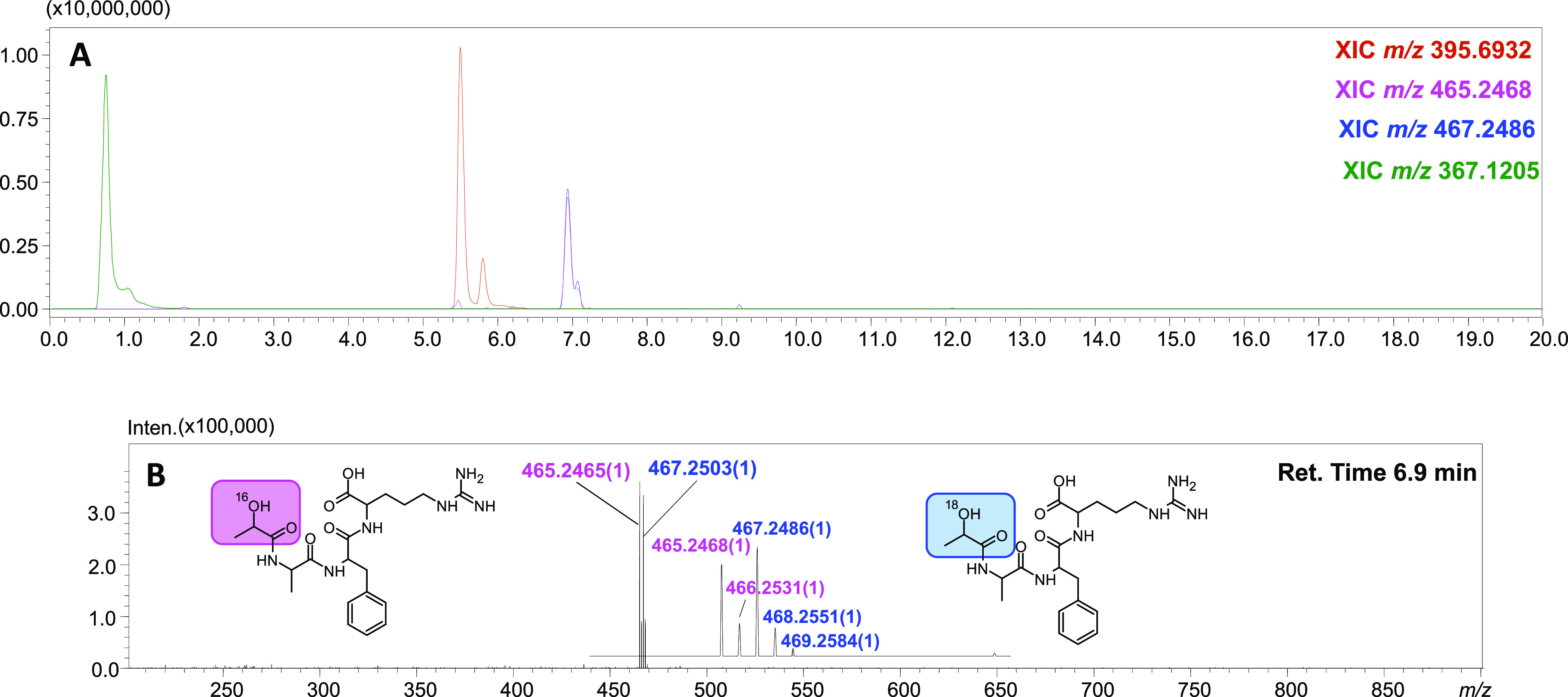
LC–MS chromatogram of the crude mixture after reaction of H-AAFR-OH with lactose and treatment with an equimolar mixture of H_2_^18^O and H_2_^16^O in the presence of NaBH_4_ (A); ESI-MS spectrum for a retention time of 6.9 min with the zoom isotopic pattern for investigated compounds, identified as reduced α-hydroxyacyl (B) (the whole spectrum with identification of all signals is placed in the Supporting Information).

In our research, we utilized various analytical methods to analyze the products of the reaction between model peptides and lactose. In addition to LC-HR-MS and MS/MS, we employed chemical methods based on the reactivity of the carbonyl group, including isotopic exchange combined with NaBH_4_ reduction and selective reaction with a hydroxylamine derivative immobilized on a solid support. The products of these reactions were subsequently analyzed by LC–MS/MS. In the first approach, we reacted the model peptide H-AAFR-OH with lactose and then treated the crude mixture with an equimolar mixture of H_2_^18^O and H_2_^16^O before reducing it with NaBH_4_. The resulting product was analyzed using LC–MS/MS, and the chromatogram presented in Figure 4 (whole spectrum in Figure S14) clearly shows two peaks with the same retention time at 6.9 min. The ESI-MS spectrum revealed two equal intensity signals at *m*/*z* 465.2468 and 467.2486, corresponding to the reduced α-ketoacyl group bearing ^16^O and ^18^O atoms, respectively. This result is similar to that obtained previously for a product of threonine oxidation^33^ and confirms the presence of a carbonyl group susceptible to isotopic exchange of oxygen atoms.

In addition to the signals corresponding to the reduced carbonylated peptide, the presented chromatogram also shows signals of reduced lactose (*m*/*z* 367.1205) and reduced α-lactosylated analog of H-AAFR-OH (*m*/*z* 395.6932).

In the second approach, we used the hydroxylamine derivative attached to the polymer, recently developed by our group^26^ for the enrichment of samples in carbonylated peptides and other compounds with reactive carbonyl groups, to confirm the presence of a deamination product. The mixture of compounds obtained after the reaction of peptides (H-AFAK-NH_2_ or H-RAKAFKA-NH_2_) with lactose was reacted with hydroxylamine immobilized on resin according to the protocol placed in Section 2. After removing products from the resin, the mixture was analyzed by LC–MS/MS. The obtained results are presented in Figures S15 and S16 (see the Supporting Information). The LC–MS chromatogram of the crude mixture after reaction with functionalized resin showed four main signals corresponding to the unreacted linker (*m*/*z* 361.1931), oxime with free lactose (*m*/*z* 685.3029), oxime with a deaminated product possessing a lactose moiety attached to the side chain of lysine residue Pyr-FAK(Lac)-NH_2_ (*m*/*z* 550.7675; charge 2+), and oxime with the deamination product Pyr-FAK-NH_2_ (*m*/*z* 388.7113; charge 2+) (Figure S15A–C,E). The oxime products were subjected to fragmentation experiments to confirm the sequence of analyzed compounds and the modification sites (Figure S15D,F). The fragmentation spectrum of oxime with a deaminated product is dominated by the characteristic furylium ion formed from the lactose moiety. The strong evidence proving the structure of the analyzed product is the signal of the product without a lactose moiety (*m*/*z* 388.7103; charge 2+) but decorated with a hydroxylamine derivative. Similar results were obtained for deaminated products of H-RAKAFKA-NH_2_ after reaction with lactose (Figure S16). The LC–MS spectrum revealed the chromatogram containing three double signals having the same retention time at *m*/*z* 377.884 (3+), 485.9208 (3+), and 593.9572 (3+) corresponding to a deaminated peptide without a lactose moiety, with one and two units of lactose attached to the side chain of lysine and arginine residues. In some cases, depending on applied gradient methods, the signals were separated but still possessed the same *m*/*z* value. This phenomenon was described by Lavrynenko et al.,^34^ explaining this behavior by the presence of a double bond between N and C in the structure of the steroid, resulting in two possible stereoisomers, *E* and *Z*.

Therefore, it was proven that reaction with lactose results in the formation of an Amadori product on the Lys moiety, while in the case of the N-terminal amino acid residue, the initially formed Amadori product is degraded to a ketoacyl moiety. The conversion of the N-terminal amino acid to keto acid was reported previously by Meltretter et al.^23^ A similar product was reported in processed milk. The authors reported on the formation of N-terminal ketoamide from the Leu residue.^24^ This result was explained by direct oxidation of the amino group or by a reaction caused by the dicarbonyl compound formed by the decomposition of the Amadori product or directly by sugar degradation. The authors took into consideration the mechanism reported previously by Akagawa et al.^35^ who demonstrated deamination of an ε-amino group in lysine, resulting in the formation of semiglutamic aldehyde. This reaction requires a Cu^2+^ ion as a catalyst. However, our model experiments indicate that there is a distinct difference between the reactivity of α- and ε-amino groups. The ε-NH_2_ group forms a stable product of Amadori rearrangement, and under the conditions applied in our experiment, there is no significant oxidation or dehydration of reaction products. On the other hand, the α-amino group undergoes an almost quantitative conversion to the keto group without Cu^2+^ or any similar catalyst. It should also be noted that the synthetic model with an acetylated α-amino group exclusively formed an Amadori product on the lysine moiety (Figure S1).

Therefore, a reaction of deamination of N-terminal amino acids is initiated by the formation of the imine with the aldehyde group of the reducing sugar. Imine **1** rearranges to imine **2**, which finally can undergo hydrolysis, producing α-ketoacyl derivative **3**. A possibility of such rearrangement finds support in a previous study.^36^ Another possible mechanism is based on the Strecker degradation. According to this mechanism, imine **1**, after rearrangement to enaminol **4**, is oxidized to the dicarbonyl imine **5**, which is susceptible to the rearrangement analog to the Strecker degradation and gives imine **6**. The last compound on hydrolysis gives the α-keto carboxylic acid amide **3**.

**Scheme 1:**
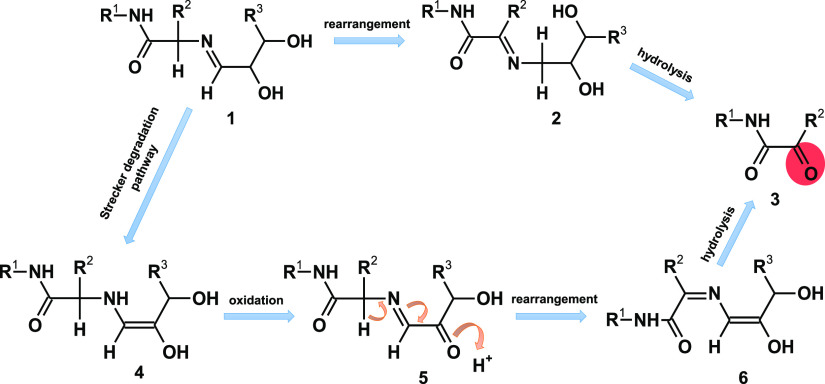
Proposed Mechanism of Deamination of N-Terminal Amino Acid in a Peptide in the Presence of a Reducing Sugar

To distinguish between the Strecker-based mechanism and direct imine isomerization, we decided to compare the reaction in the presence and absence of oxygen. A lyophilized mixture of lactose and H-AKAF-NH_2_ peptide was incubated in an open vial at 120 °C, while an identical sample was heated (at the same temperature) in a vacuum-sealed ampulla. The results of these experiments (presented in the Supporting Information) were the same, indicating that the oxidization of the sugar moiety is not a necessary step of conversion of the N-terminal amino acid residue to the corresponding ketoamide. This suggests that direct isomerization resulting from proton transfer is more likely than the Strecker-based mechanism, which requires oxidation (Scheme 1).

Based on this hypothetical mechanism, we conclude that such a reaction should be possible not only for lactose but also for other reducing sugars. Therefore, we tested the reaction of free α-amino group-containing peptides with glucose. The temperature applied in this experiment was higher than during standard solid-phase glycation because, in the previous condition, we did not observe the deamination of the α-amino group and the formation of the keto acid derivative.^27,28^ In these conditions, we observed a reaction similar to that observed for lactose—the ε-amino group forms an Amadori product, while the α-amino group undergoes oxidative deamination. We established two factors responsible for deamination: acidic properties of α-hydrogen in Schiff base formed from the N-terminal amino acid moiety and high temperature. The high temperature was applied in our experiments concerning the formation of Amadori products by lactose because of the lower reactivity of lactose as compared to glucose. High-temperature glycation was carried out at a relatively low temperature (80 °C for 20 min),^37^ while for lactose, we decided to increase the temperature to 120 °C and extend the reaction time. However, besides glycation/lactosylation on the lysine side chain, the oxidative deamination of the N-terminal amino group takes place in these conditions (Figures S19 and 20 and Table S1). Performing a reaction with glucose at higher-than-usual temperatures results in oxidative deamination similar to that of lactose. Moreover, the results obtained for H-AK(*Fru*)AF-OH in the presence of lactose show the decomposition of a bond between deoxyfructose and the amino group in the side chain of lysine (Figure S21). Therefore, lactosylated products were also formed and identified. For all model peptides tested in this reaction without sugar, no deamination products were observed. In the case of the sample with glucose treated at 120 °C for 4 h, the browning of the sample was noticed and the solubility of the sample in water decreased significantly. According to the literature, the advanced glycation end products can be formed after long treatment of a sample in proposed conditions. Therefore, we did not analyze all formed products but focused mostly on the deamination of the N-terminal amino acid. We also tested the model peptide without the addition of sugar. The high-temperature treatment of the peptide without a reducing sugar does not cause the oxidative deamination of the N-terminal amino acid moiety. Therefore, the presence of a reducing sugar in this reaction is crucial, which additionally supports the proposed mechanism.

In this study, we focused on optimizing the reaction conditions in the solid phase to produce the deamination product in peptides that contain a free amino group. Our study is not limited to LC–MS/MS analysis combined with bioinformatics, which is a generally accepted practice for modification analysis. Additionally, we performed reduction combined with isotope exchange and utilized the reactivity of the carbonyl group in nucleophilic addition reactions. Our experiments suggest that the deamination reaction is a general process and takes place at the N-terminal amino acid at a temperature of approximately 120 °C in the presence of a reducing sugar. The present study proposes a mechanism for the formation of carbonylated peptides through a direct reaction between reducing sugars (lactose and glucose) and N-terminal amino acid moieties. Since every protein contains only one N-terminal amino acid, this reaction does not seem relevant in food systems where oxidative deamination affects less than 1% of amino acids in the protein. However, the reaction discussed herein may be a significant modification in partially hydrolyzed hypoallergenic infant formulas, which, depending on the hydrolysis level, may contain a higher percentage of α-amino groups.

## Supporting Information Available

ESI-MS and LC-MS chromatograms of products after reaction with lactose in different reaction conditions (PDF)
