# Supplementary material for: High-Temperature, Solid-Phase Reaction of α-Amino Groups in Peptides with Lactose and Glucose: An Alternative Mechanism Leading to an α-Ketoacyl Derivative
Source: J Agric Food Chem. 2023 Mar 31;71(14):5796–803. doi: 10.1021/acs.jafc.3c00821 (PMC10103172; doi:10.1021/acs.jafc.3c00821)
Supplement: Supplementary file 1 — jf3c00821_si_001.pdf [file jf3c00821_si_001.pdf]

Supporting Information

High-temperature, solid-phase reaction of α-amino groups in peptides with lactose and glucose – an alternative mechanism leading to α-ketoacyl derivative

Monika Kijewska*, Michalina Zawadzka, Piotr Stefanowicz*

Faculty of Chemistry, University of Wrocław, Joliot-Curie 14, 50-383 Wrocław, Poland

Corresponding authors*: Monika Kijewska (ORCID 0000-0001-6227-7169),
Piotr Stefanowicz (ORCID 0000-0001-9581-2359)
Faculty of Chemistry, University of Wrocław, F. Joliot-Curie 14, 50-383 Wrocław, Poland, Fax: +48-71-3282348, Tel.: +48-71-3757250, +48-71-3757213
E-mail: monika.kijewska@chem.uni.wroc.pl
E-mail: piotr.stefanowicz@chem.uni.wroc.pl

LC-MS analysis

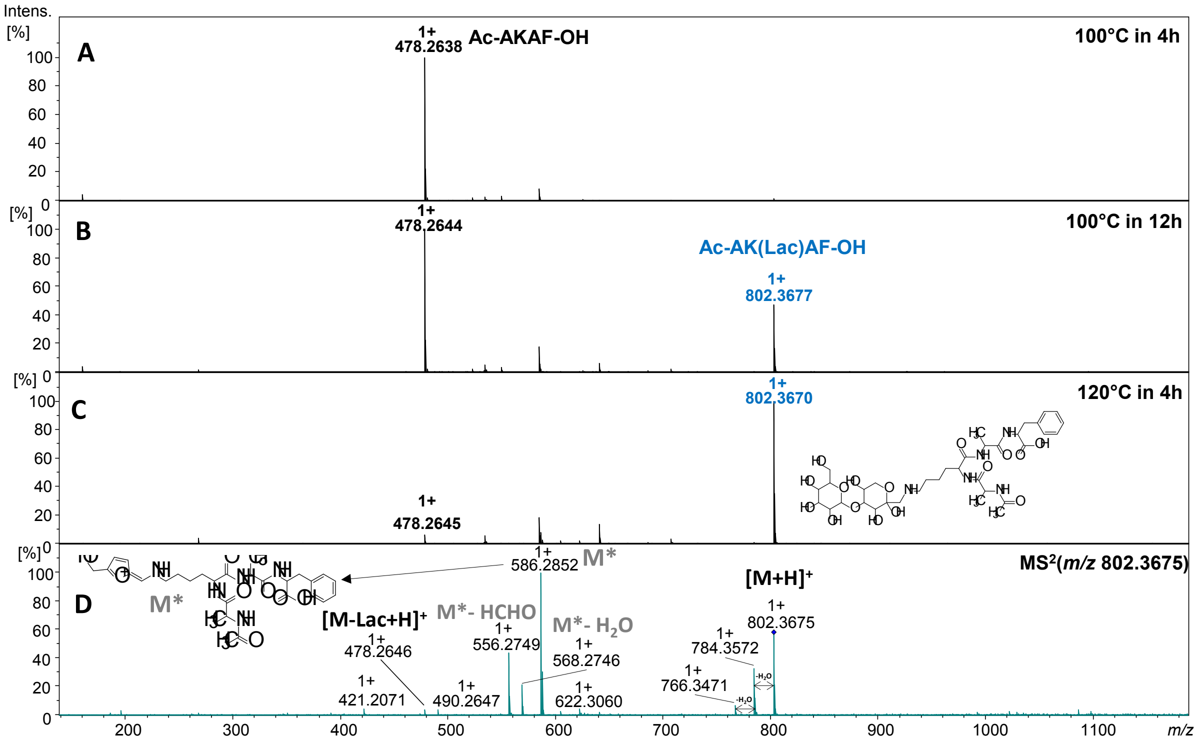

Fig. S1 ESI-MS spectra for the crude mixture of model peptide Ac-AKAF-OH after reaction with lactose in different conditions (A-C); ESI-MS spectrum for Ac-AK(Lac)AF-OH; parent ion m/z 802.3675collision energy: 25eV (D).

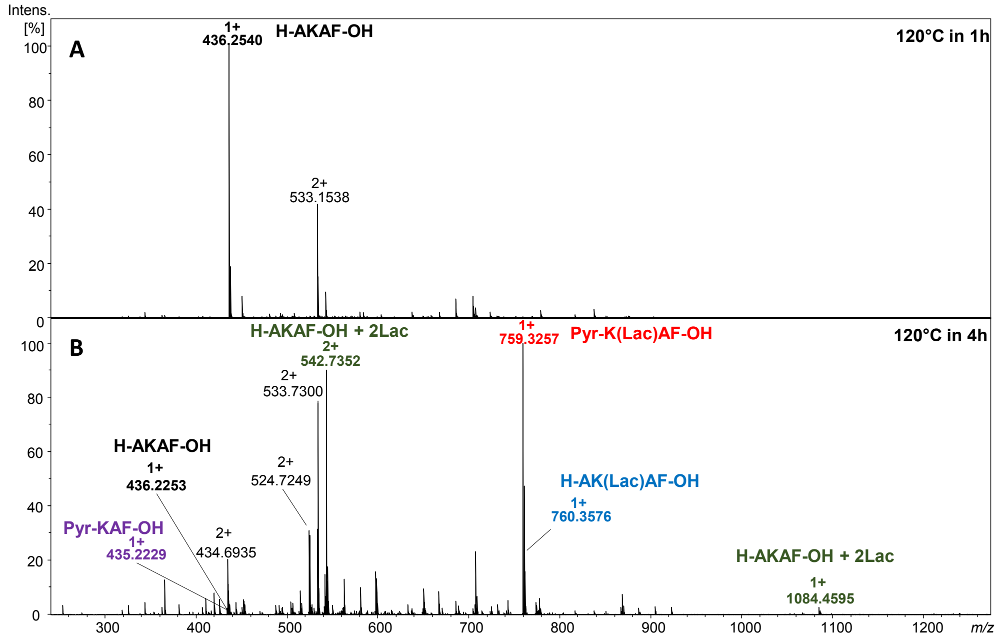

Fig. S2 ESI-MS spectra for the crude mixture of model peptide H-AKAF-OH after reaction with lactose in different conditions (A-B).

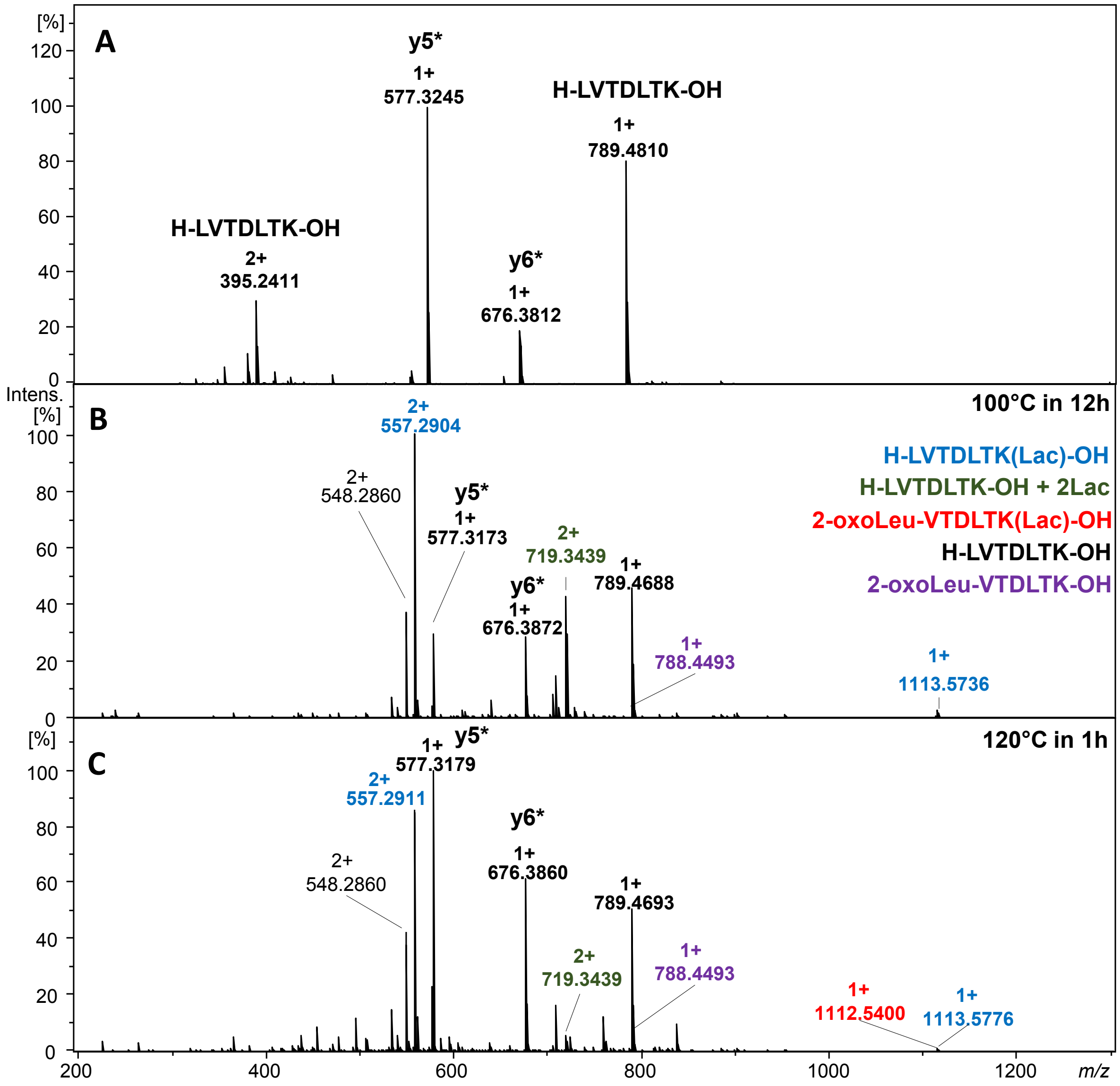

Fig. S3 ESI-MS spectrum of model peptide H-LVTDLTK-OH (* - fragmentation ions forming in ion source) (A); ESI-MS spectra for the crude mixture of model peptide H-LVTDLTK-OH after reaction with lactose in different conditions (B-C).

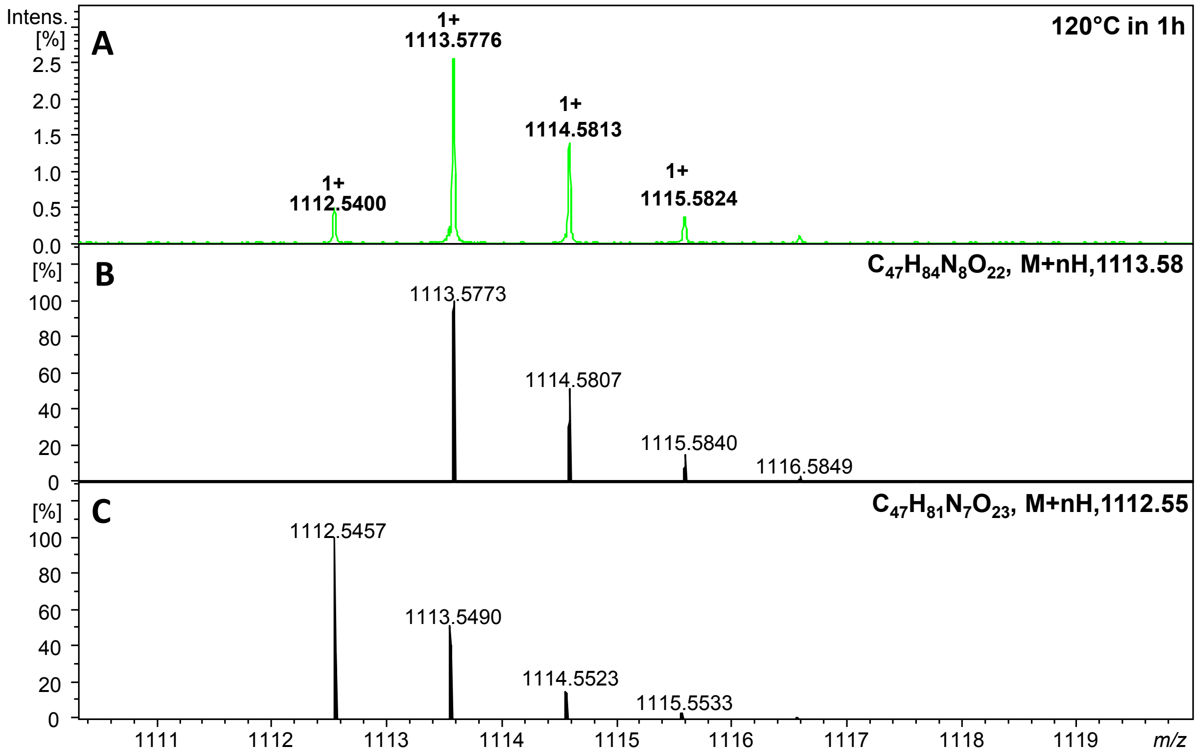

Fig. S4 Expanded range of ESI-MS spectrum for H-LVTDLTK-OH after reaction with lactose (A); simulated ESI-MS spectrum for H-LVTDLTK(Lac)-OH (B); simulated ESI-MS spectrum for 2-oxoLeu-VTDLTK(Lac)-OH (C).

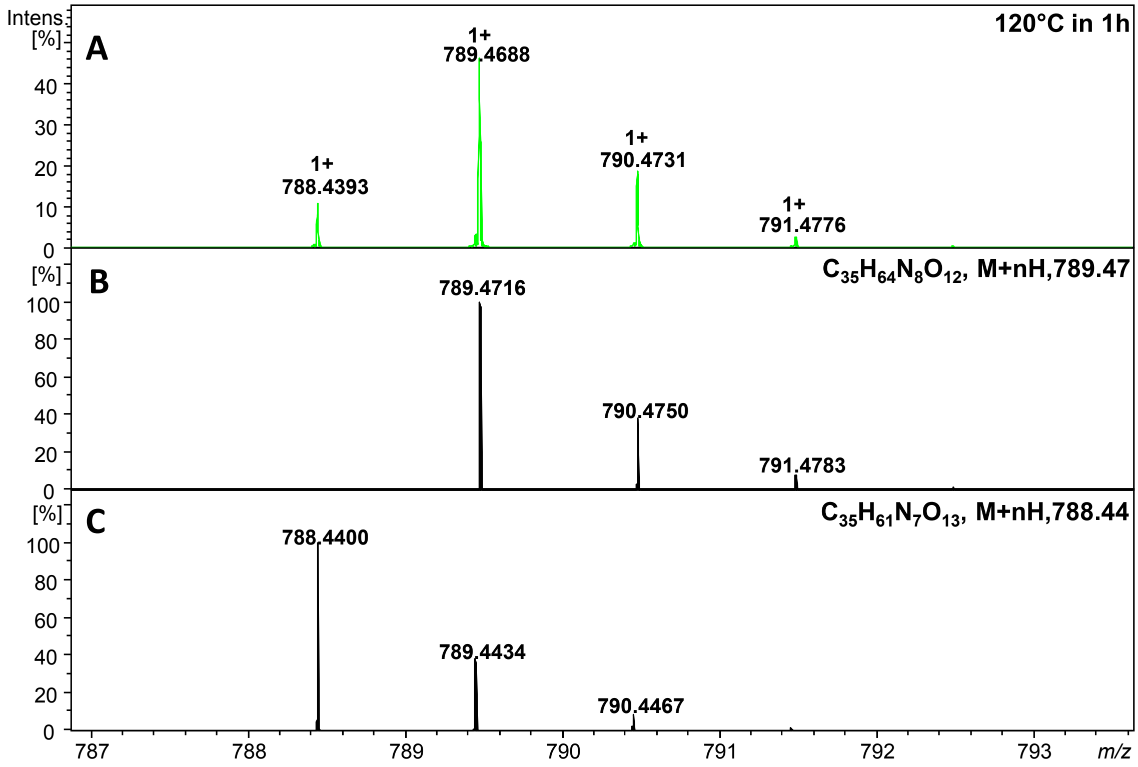

Fig. S5 Expanded range of ESI-MS spectrum for H-LVTDLTK-OH after reaction with lactose (A); simulated ESI-MS spectrum for H-LVTDLTK-OH (B); simulated ESI-MS spectrum for 2-oxoLeu-VTDLTK-OH (C).

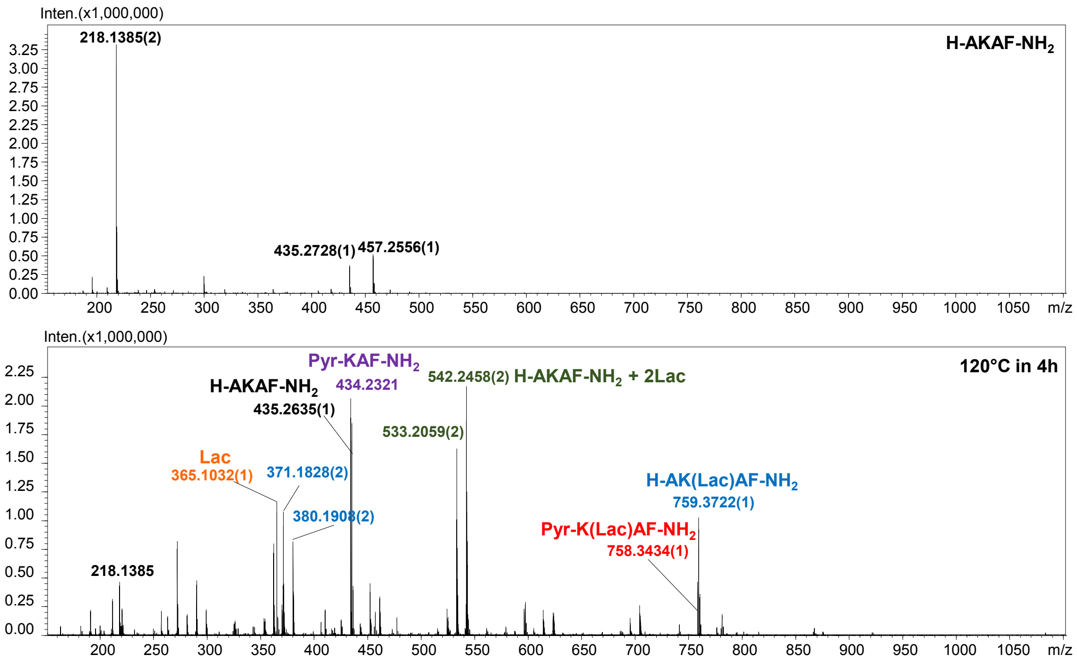

Fig. S6 ESI-MS of model peptide H-AKAF-NH_2 before and after reaction with lactose (*reaction conditions*: 120°C at 4 h).

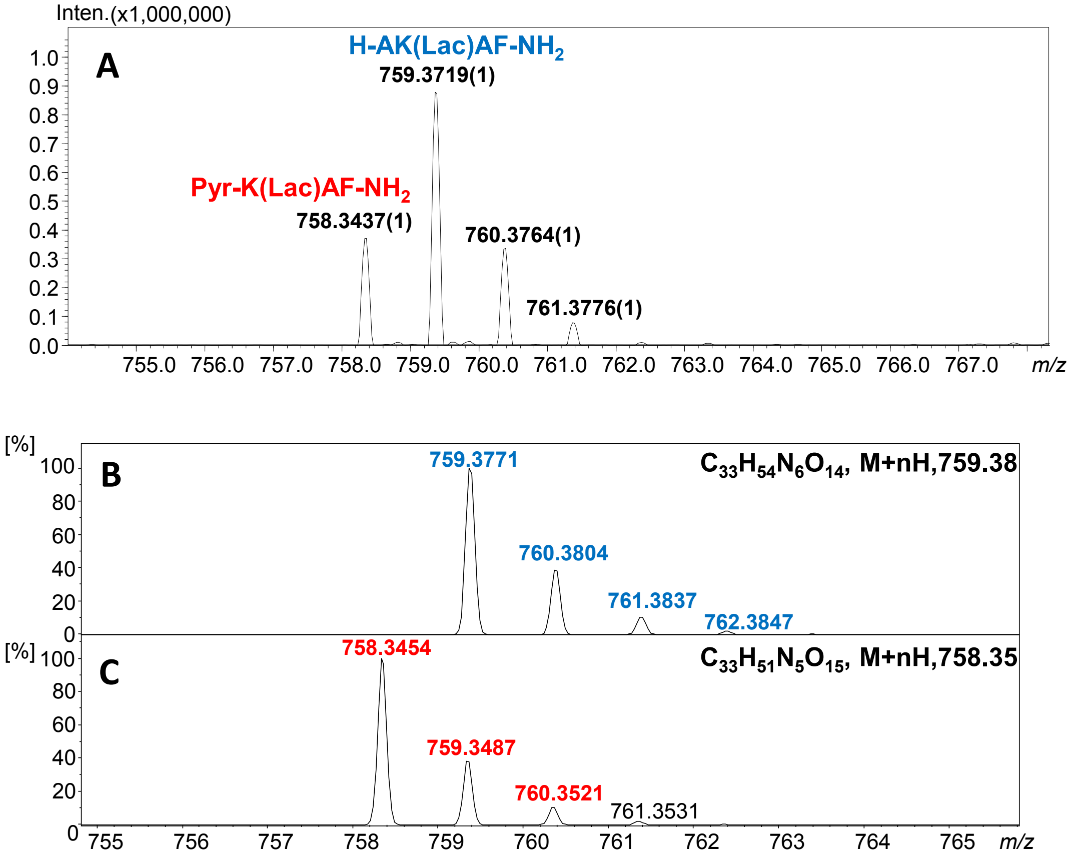

Fig. S7 Expanded range of ESI-MS spectrum for H-AKAF-NH_2 after reaction with lactose (A); simulated ESI-MS spectrum for H-AK(Lac)AF-NH_2 (B); simulated ESI-MS spectrum for Pyr-K(Lac)AF-NH_2 (C).

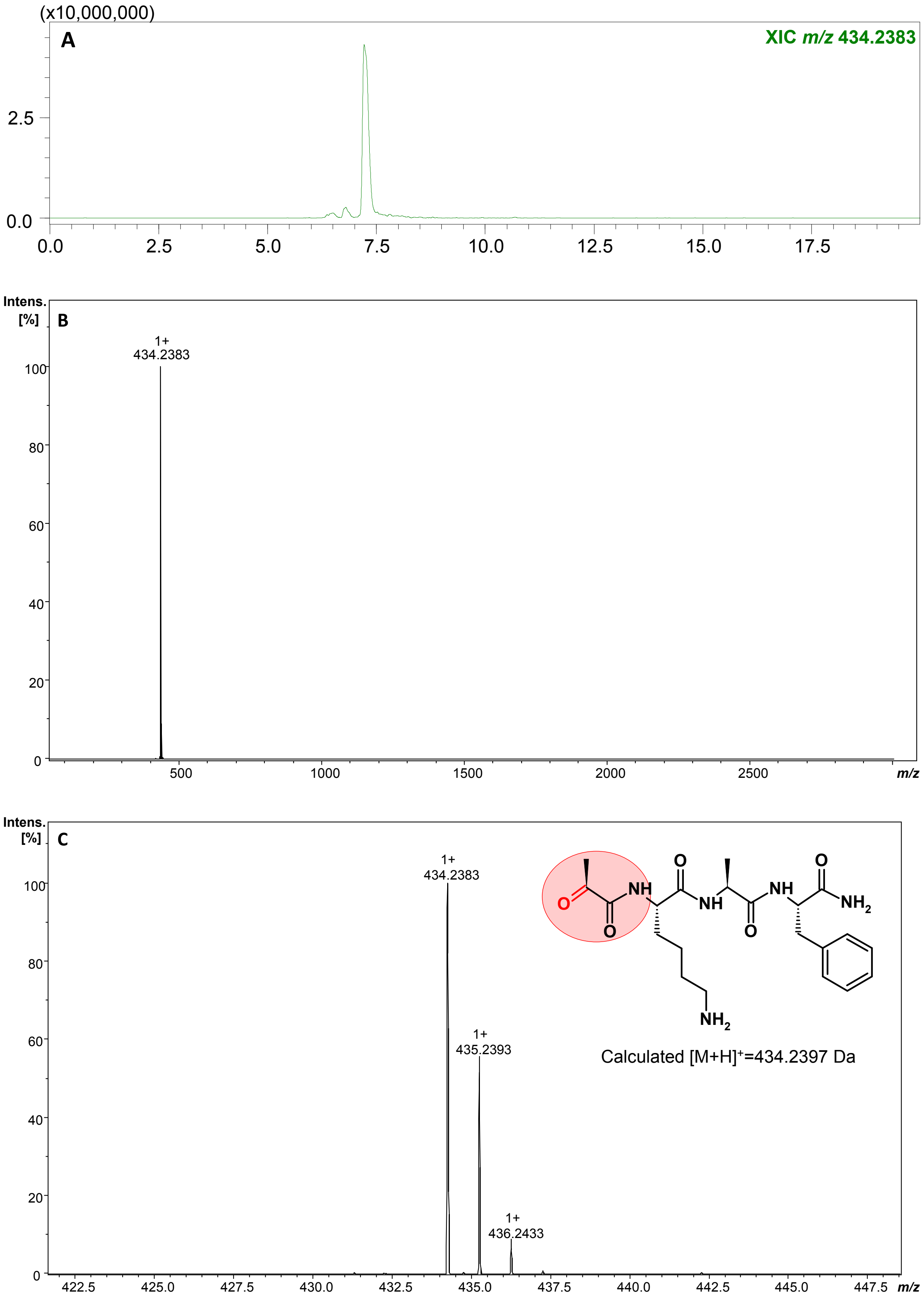
(x10,000,000)
A
XIC m/z 434.2383
2.5
0.0
0.0
2.5
5.0
7.5
10.0
12.5
15.0
17.5
Intens.
[%]
B
1+
434.2383
100
80
60
40
20
0
500
1000
1500
2000
2500
m/z
Intens.
[%]
C
1+
434.2383
1+
435.2393
1+
436.2433
100
80
60
40
20
0
422.5
425.0
427.5
430.0
432.5
435.0
437.5
440.0
442.5
445.0
447.5
m/z
O
NH
NH
NH
NH2
NH2
Calculated [M+H]+=434.2397 Da

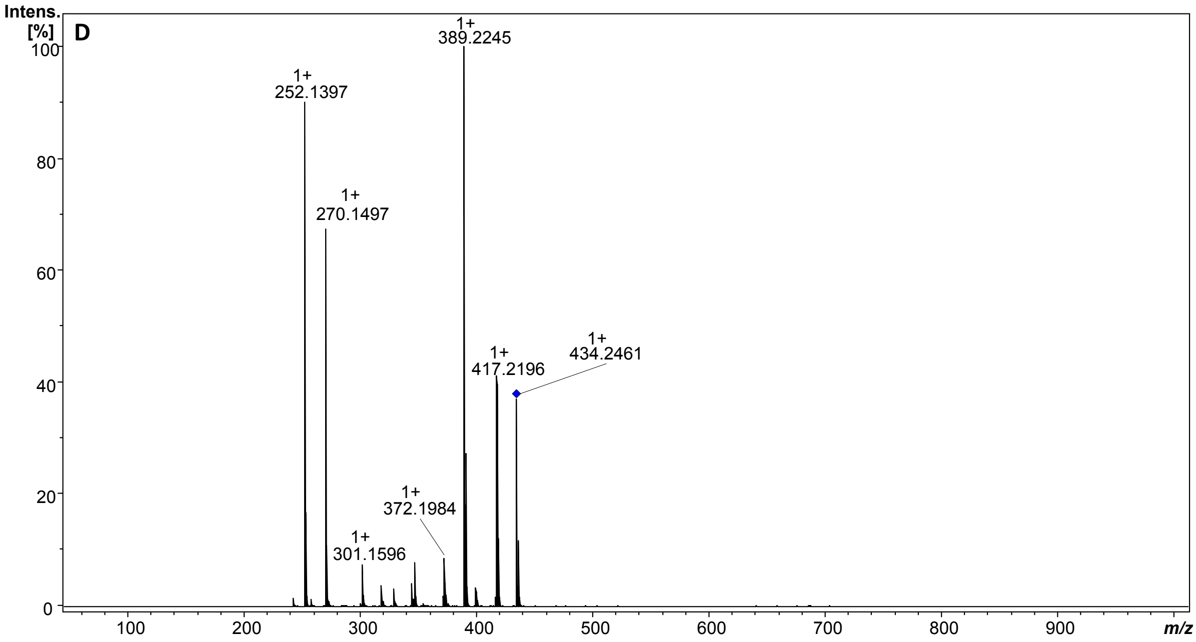

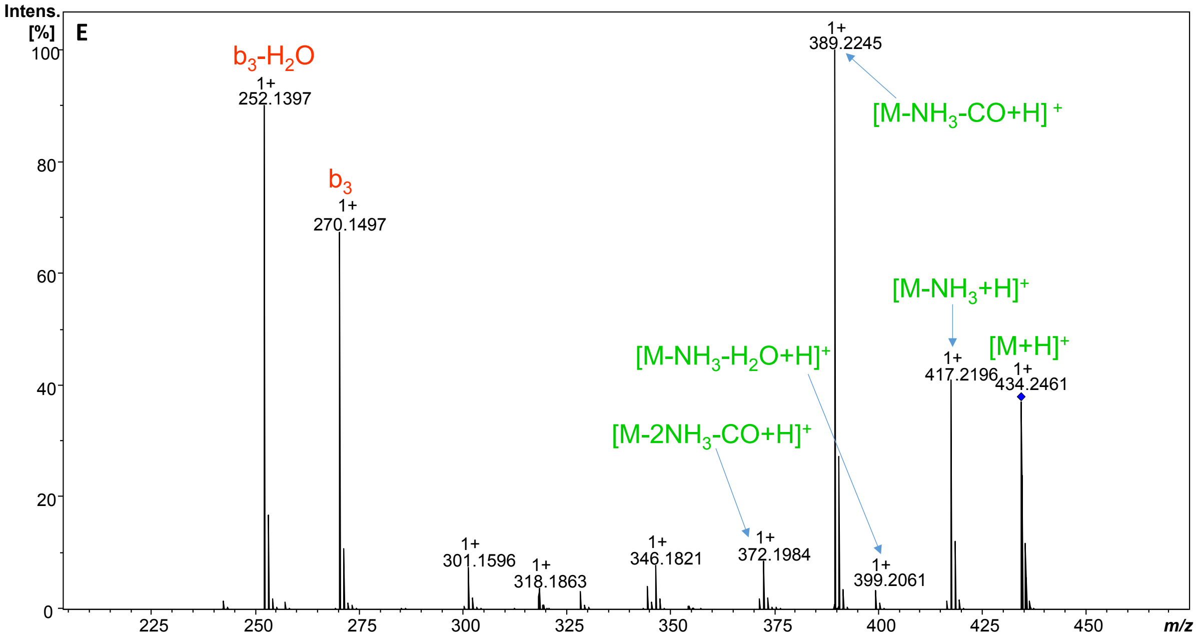

Fig. S8 LC-MS chromatogram of synthetic Pyr-KAF-NH_2 (A); ESI-MS analysis (B, C - zoom); ESI-MS/MS analysis for parent ion at *m/z* 434.2321, collision energy 25 eV (D, E - zoom)

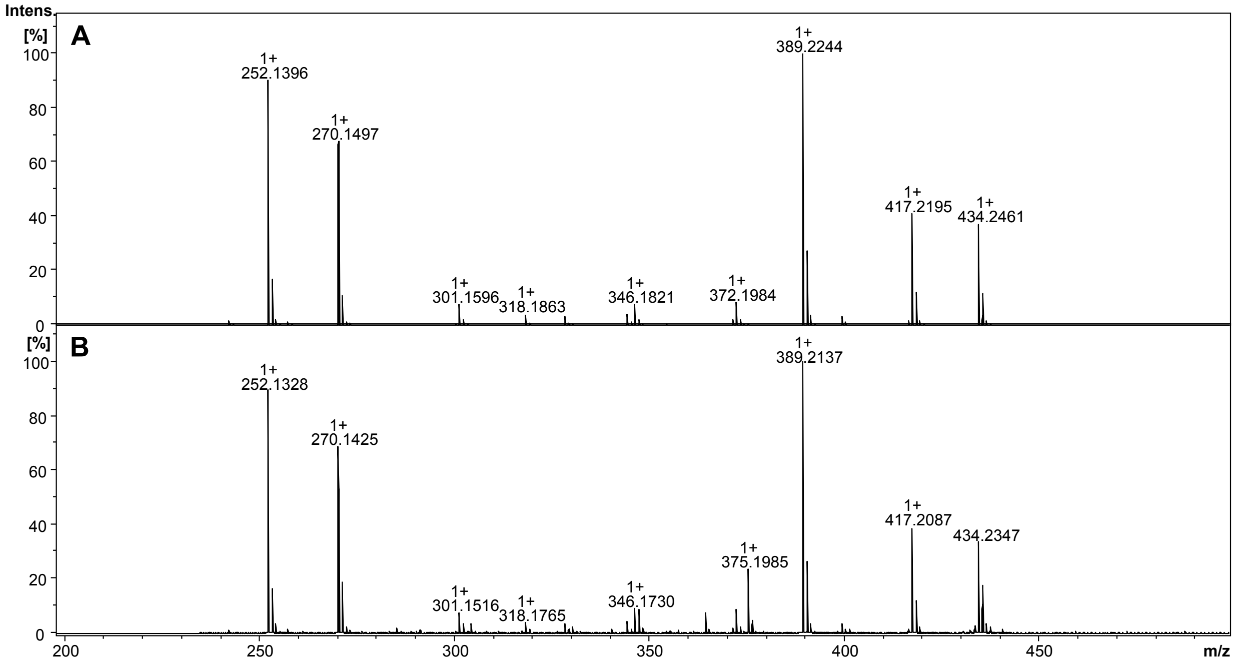

Fig. S9 Comparison of fragmentation spectra of synthetic Pyr-KAF-NH_2 (A) and deaminated product (Pyr-KAF-NH_2) obtained in the reaction of H-AKAF-NH_2 with lactose (B)

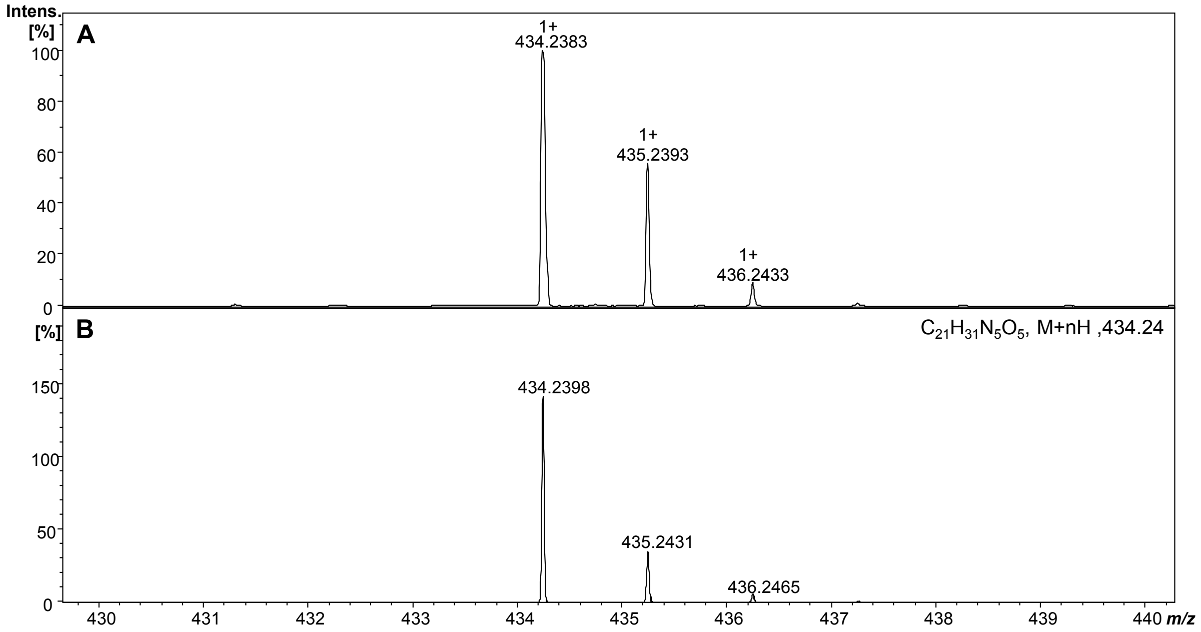

Fig. S10 ESI-MS spectrum of Pyr-KAF-NH_2 (A) and simulated isotopic pattern for molecular formula $C_{21}H_{31}N_5O_5$ (B)

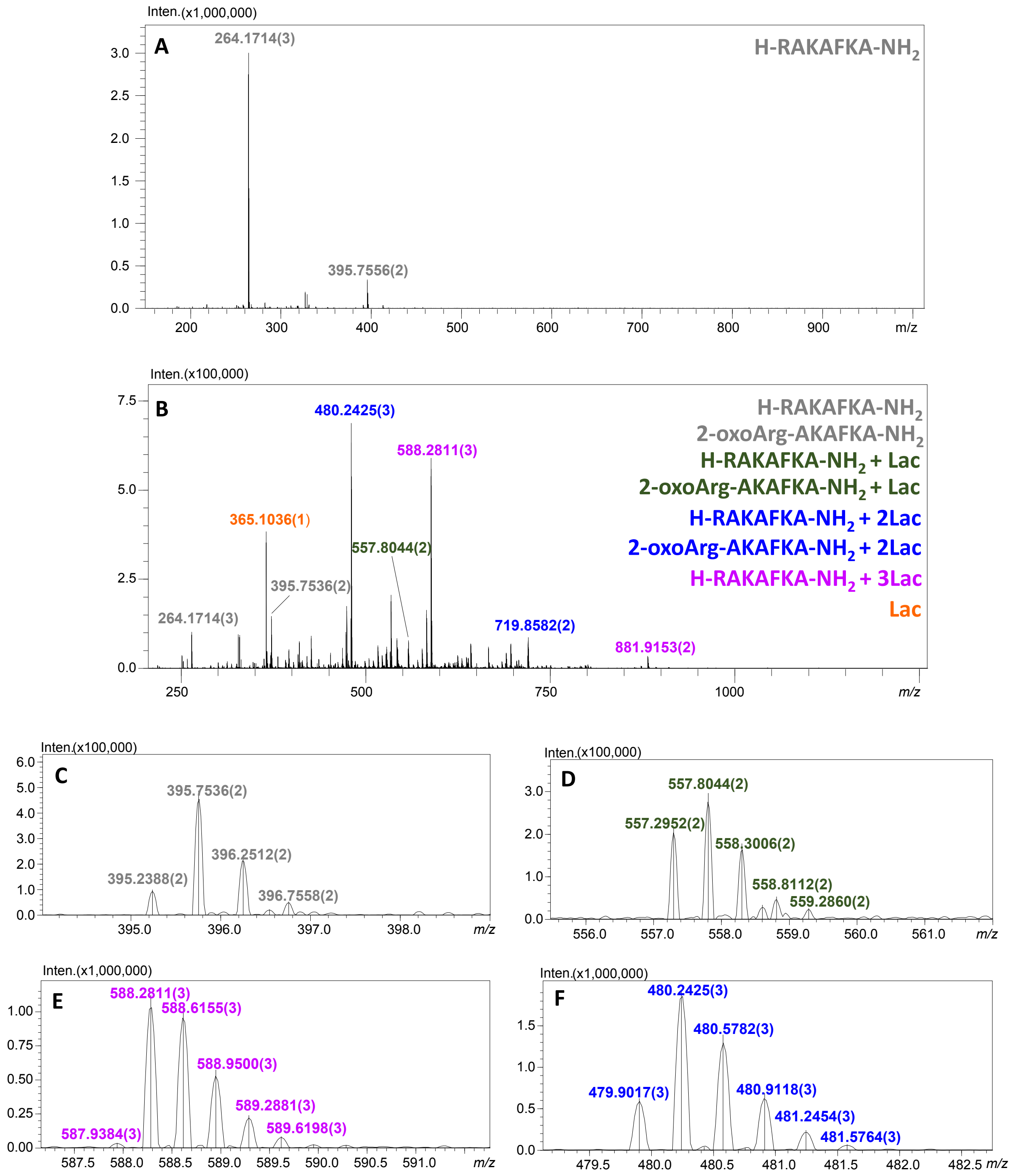

Fig. S11 ESI-MS analysis of model peptide H-RAKAFKA-NH_2 before (A) and after reaction with lactose (B). Zoom in on the appropriate spectral ranges to show the isotope distribution of the following signals: C – corresponds to a mixture of compounds: H-RAKAFKA-NH_2 and 2-oxoArg-AKAFKA-NH_2; D – corresponds to a mixture of compounds: H-RAKAFKA-NH_2 + Lac and 2-oxoArg-AKAFKA-NH_2 + Lac; E – corresponds to a mixture of compounds: H-RAKAFKA-NH_2 + 3Lac; E – corresponds to a mixture of compounds: H-RAKAFKA-NH_2 + 2Lac and 2-oxoArg-AKAFKA-NH_2 +2Lac.

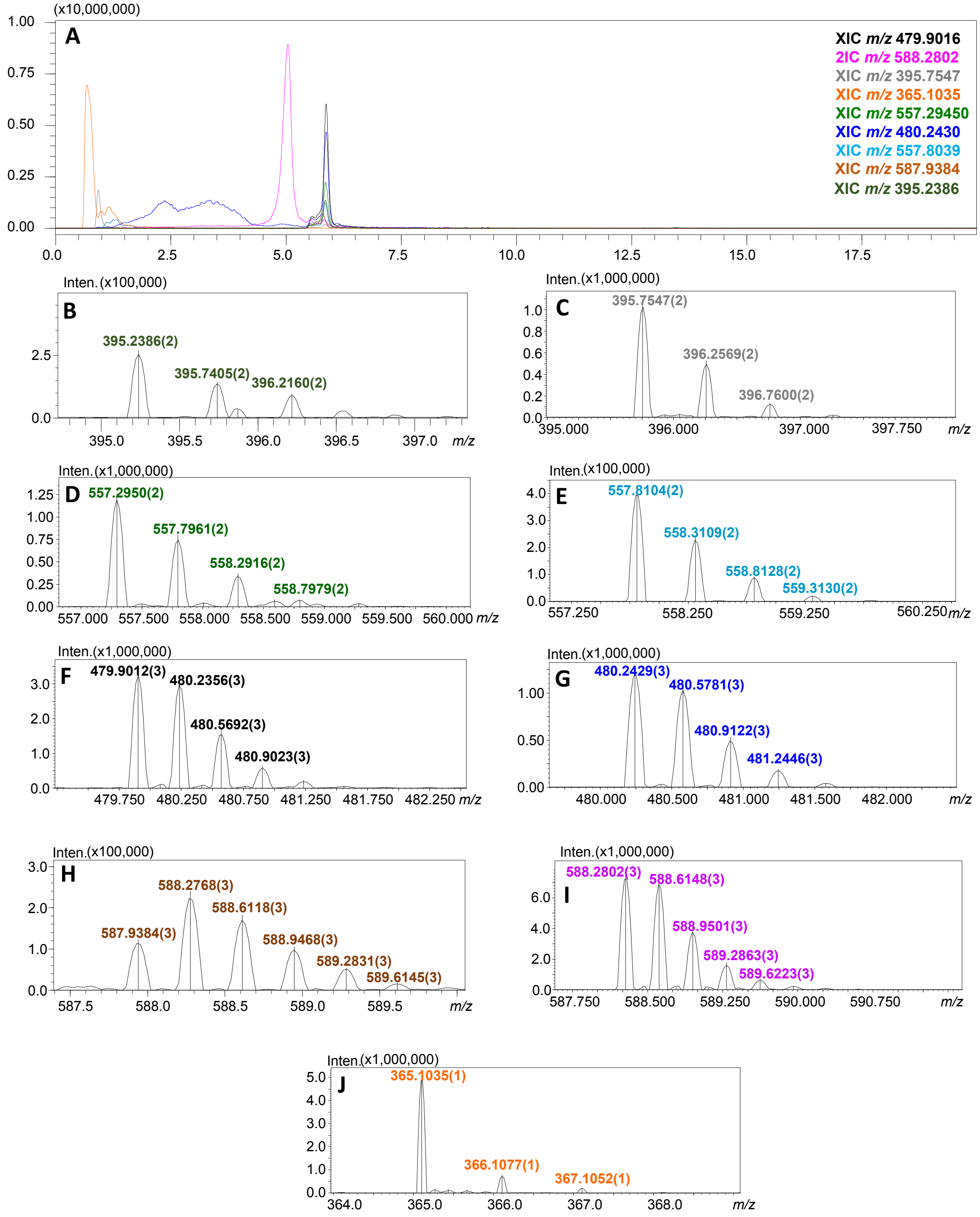

Fig. S12 LC-MS chromatogram of mixture of H-RAKAFKA-NH_2 after reaction with lactose (A); ESI-MS spectra (zoom) for identified compounds (B-J); B - ESI-MS (zoom) of deaminated analog; C - ESI-MS (zoom) of unmodified analog; D - ESI-MS (zoom) of deaminated analog with lactose; E - ESI-MS (zoom) of analog with lactose; F - ESI-MS (zoom) of deaminated analog with two lactose units; G - ESI-MS (zoom) of analog with two lactose units; H - ESI-MS (zoom) of deaminated analog with three lactose units; I - ESI-MS (zoom) of analog with three lactose units; J - ESI-MS (zoom) of lactose.

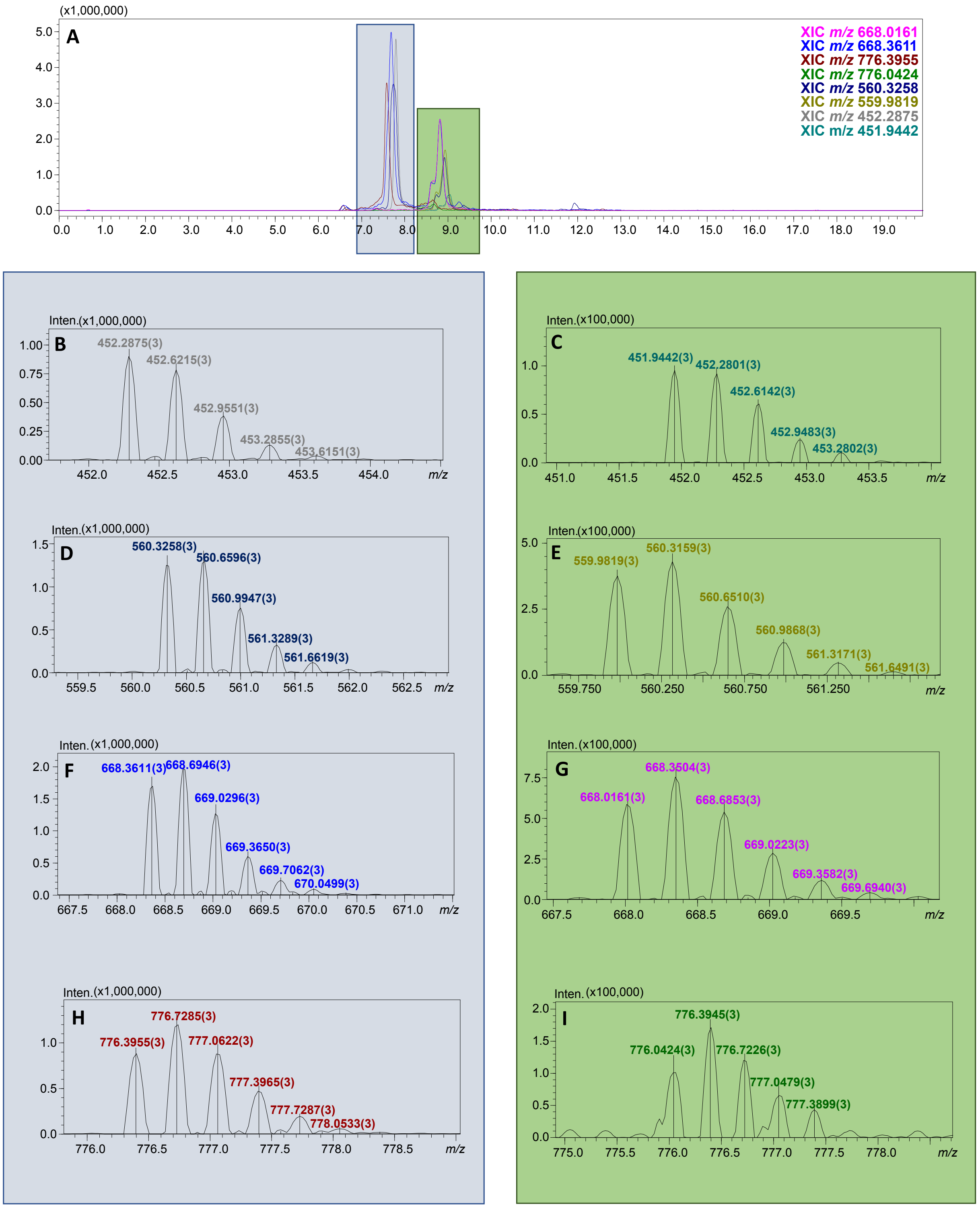

Fig. S13 LC-MS chromatogram of mixture of H-SEVLRLVKDPAK-OH after reaction with lactose (A); ESI-MS spectra (zoom) for identified compounds (B-I); B - ESI-MS (zoom) of deaminated analog; C - ESI-MS (zoom) of unmodified analog; D - ESI-MS (zoom) of deaminated analog with lactose; E - ESI-MS (zoom) of analog with lactose; F - ESI-MS (zoom) of deaminated analog with two lactose units; G - ESI-MS (zoom) of analog with two lactose units; H - ESI-MS (zoom) of deaminated analog with three lactose units; I - ESI-MS (zoom) of analog with three lactose units; (blue part – unmodified analog and lactosylated forms; green part – deaminated compounds)

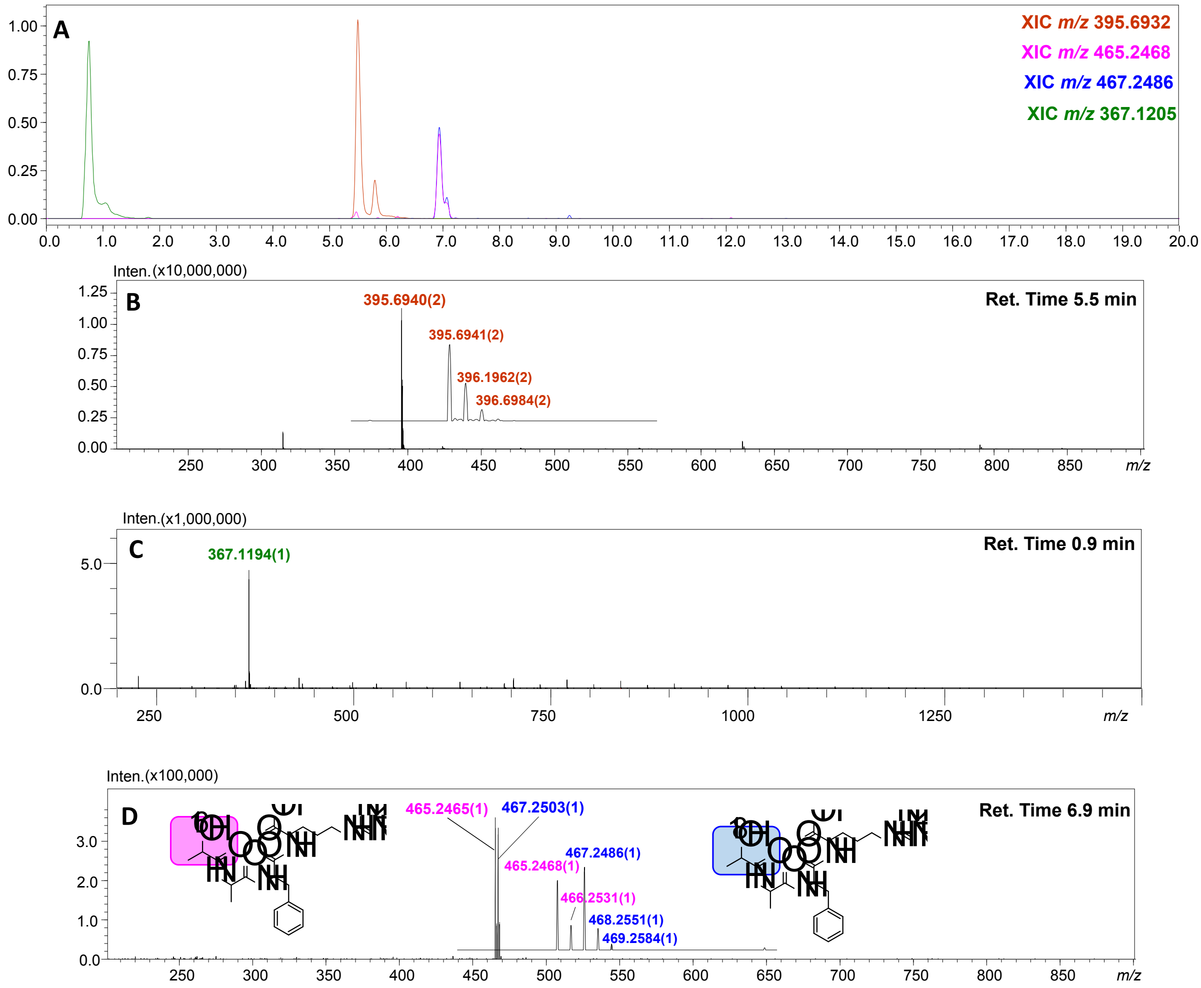

Fig. S14 LC-MS chromatogram of crude mixture of H-AAFR-OH after reaction with lactose and treated with equimolar mixture of $H_2{}^{18}O$ and $H_2{}^{16}O$ in the presence of $NaBH_4$ (A); ESI-MS spectrum for Ret. Time 5.5 min- identified as reduced α-lactosylated analog of H-AAFR-OH (B); ESI-MS spectrum for Ret. Time 0.9 min – identified as reduced lactose (C); ESI-MS spectrum for Ret. Time 6.9 min with zoom isotopic pattern for investigated compounds – identified as appropriate reduced α-hydroxyacyl (D).

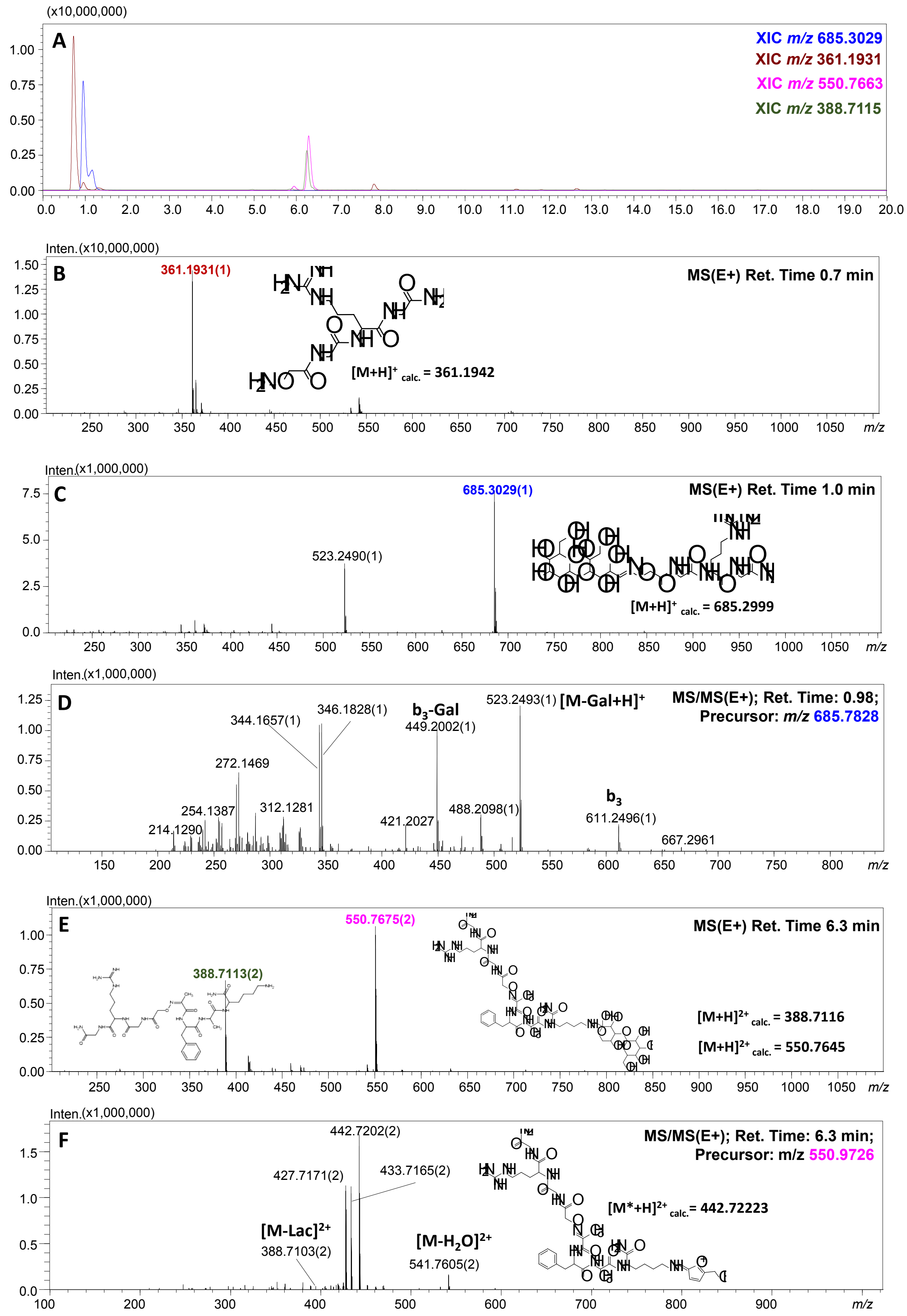

Fig. S15 LC-MS chromatogram of crude mixture after reaction with functionalized resin containing hydroxyloamine derivative (A); ESI-MS spectra of unreacted linker (B) and oxime products (C, E); ESI-MS/MS spectra of oxime products (D, F).

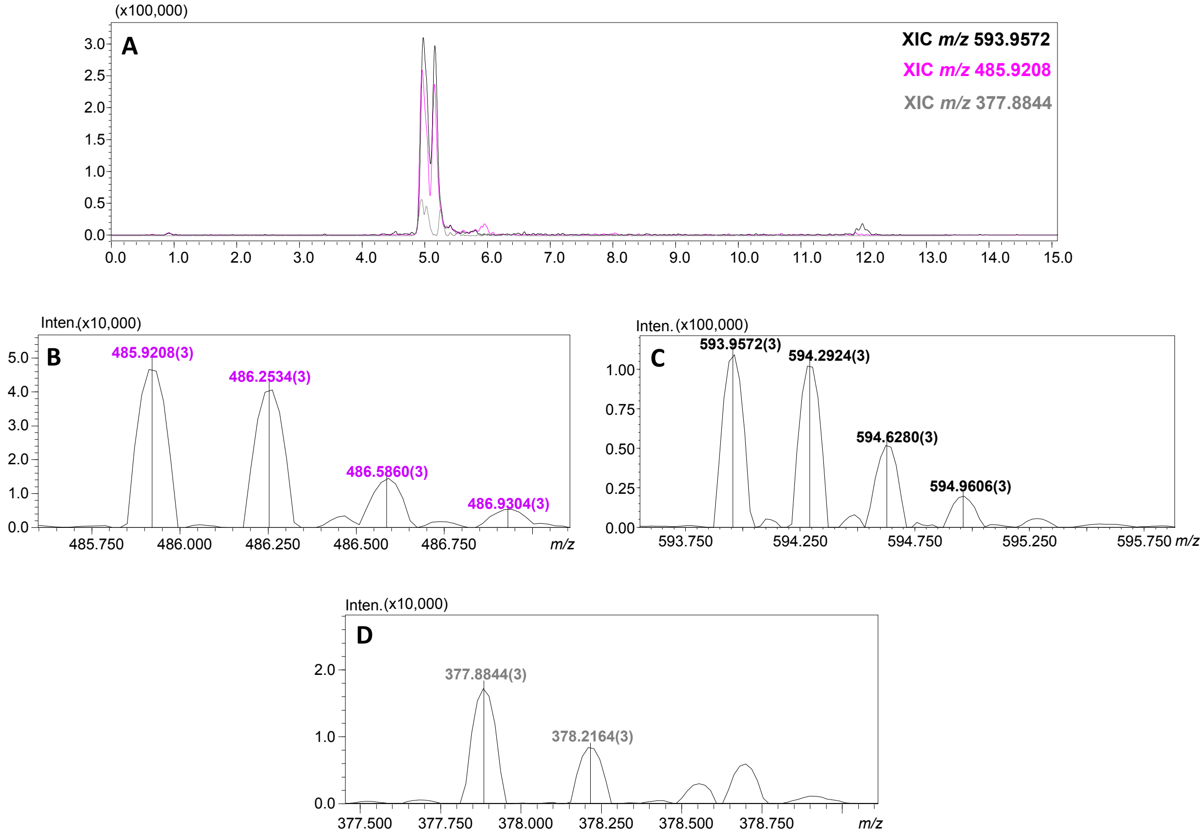

Fig. S16 LC-MS chromatogram of crude mixture (peptide H-RAKAFKA-NH_2 after reaction with lactose) after reaction with functionalized resin containing hydroxyloamine derivative (A); ESI-MS of (zoom) oxime of deaminated analog with 1 Lac (B); ESI-MS of oxime of deaminated analog with 1 Lac (C); ESI-MS of oxime of deaminated analog with 2 Lac (C); ESI-MS of oxime of deaminated analog (D).

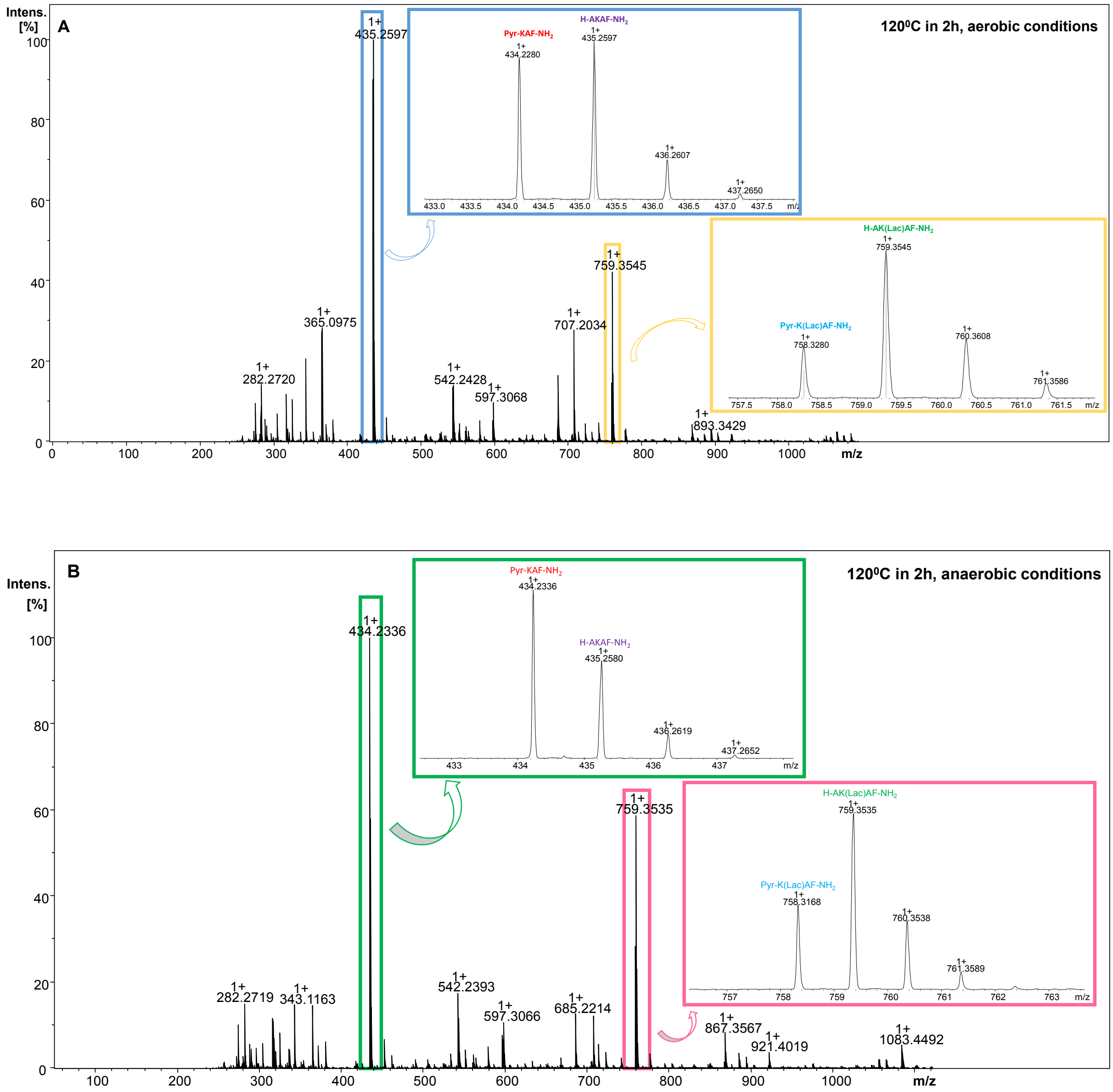

Fig. S17 ESI-MS spectra of crude mixture after reaction of H-AKAF-NH2 with lactose in conditions: A) 2h, aerobic conditions; B) 2h, anaerobic conditions

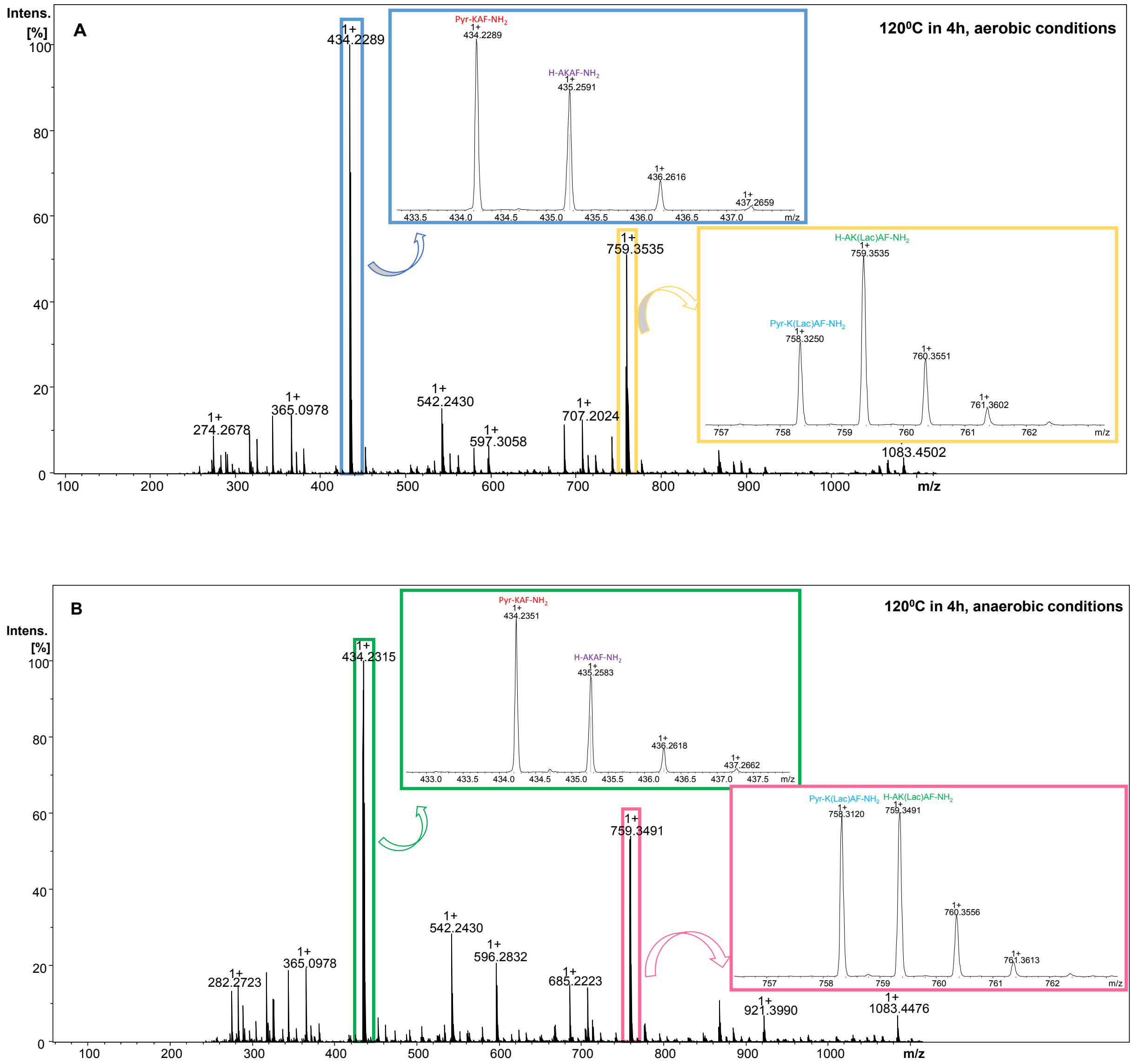

Fig. S18 ESI-MS spectra of crude mixture after reaction of H-AKAF-NH2 with lactose in conditions: A) 4h, aerobic conditions; B) 4h, anaerobic conditions

H-AKAF-NH$_2$: conditions: lactose, 120°C in 4h

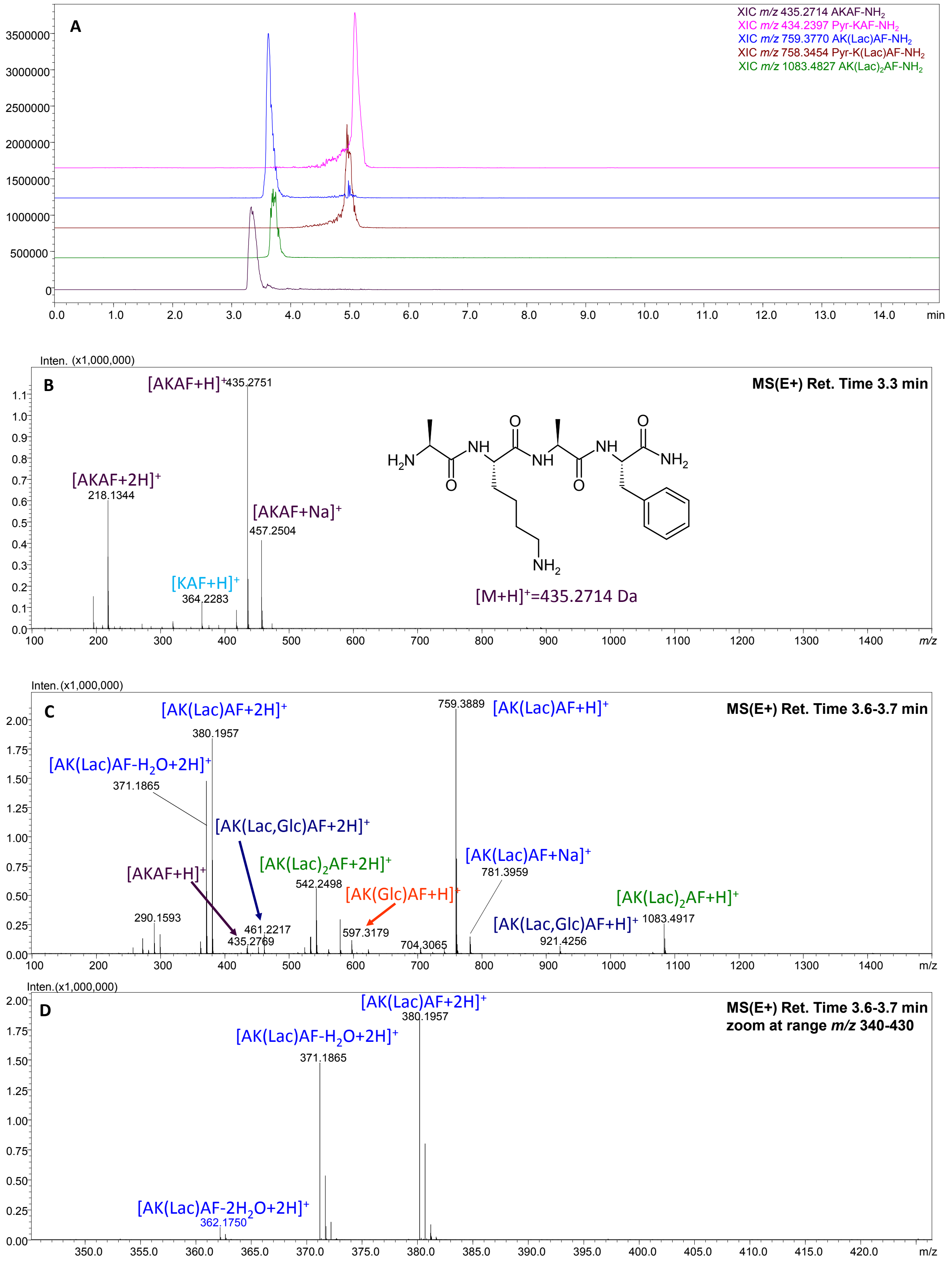

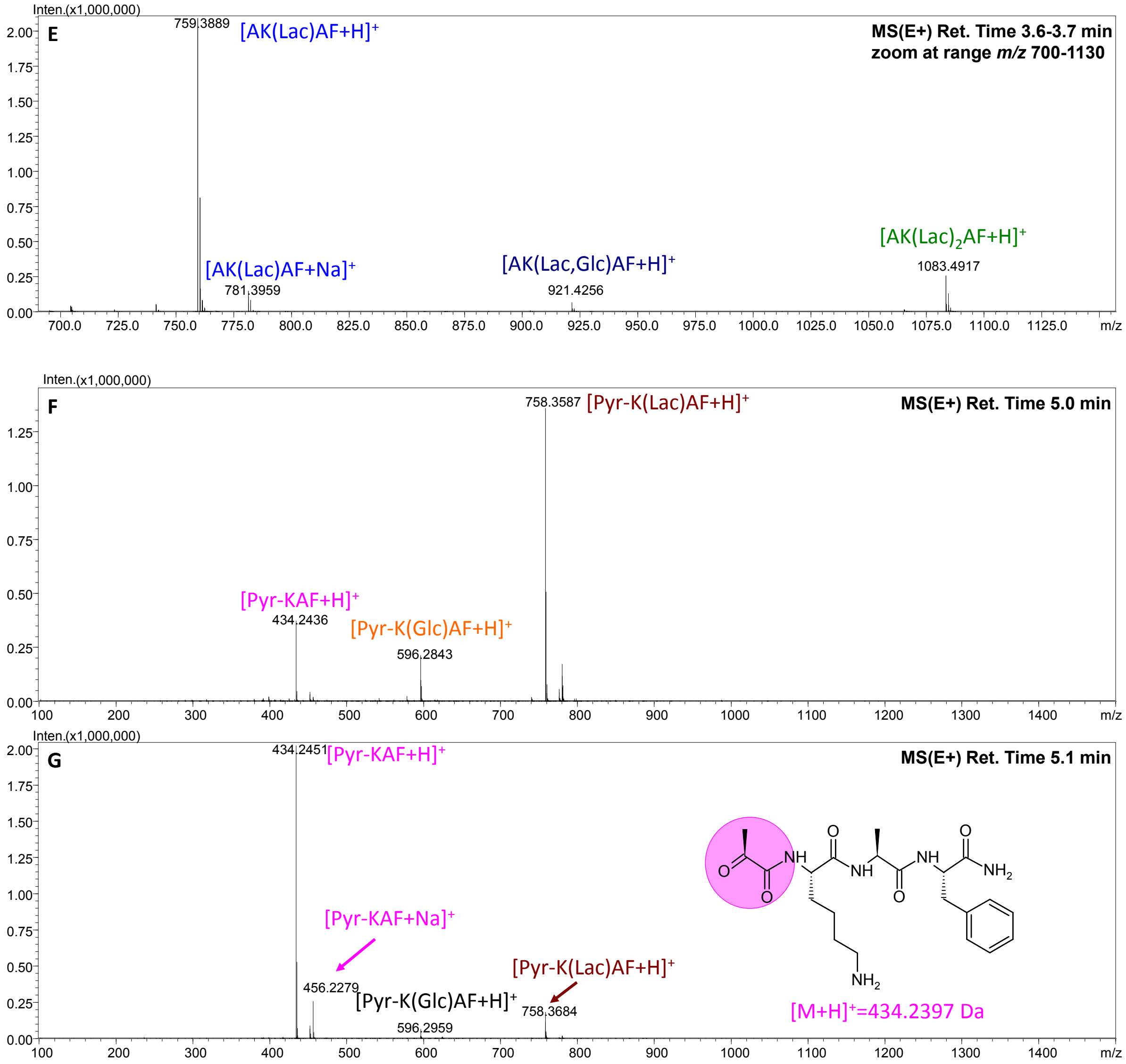

Fig. S19 LC-MS chromatogram of crude mixture of H-AKAF-NH_2 after reaction with lactose in conditions: 120°C in 4h (A); ESI-MS spectra for all identified signals on chromatogram (B-G).

H-AKAF-NH_2: conditions: glucose, 120°C in 4h

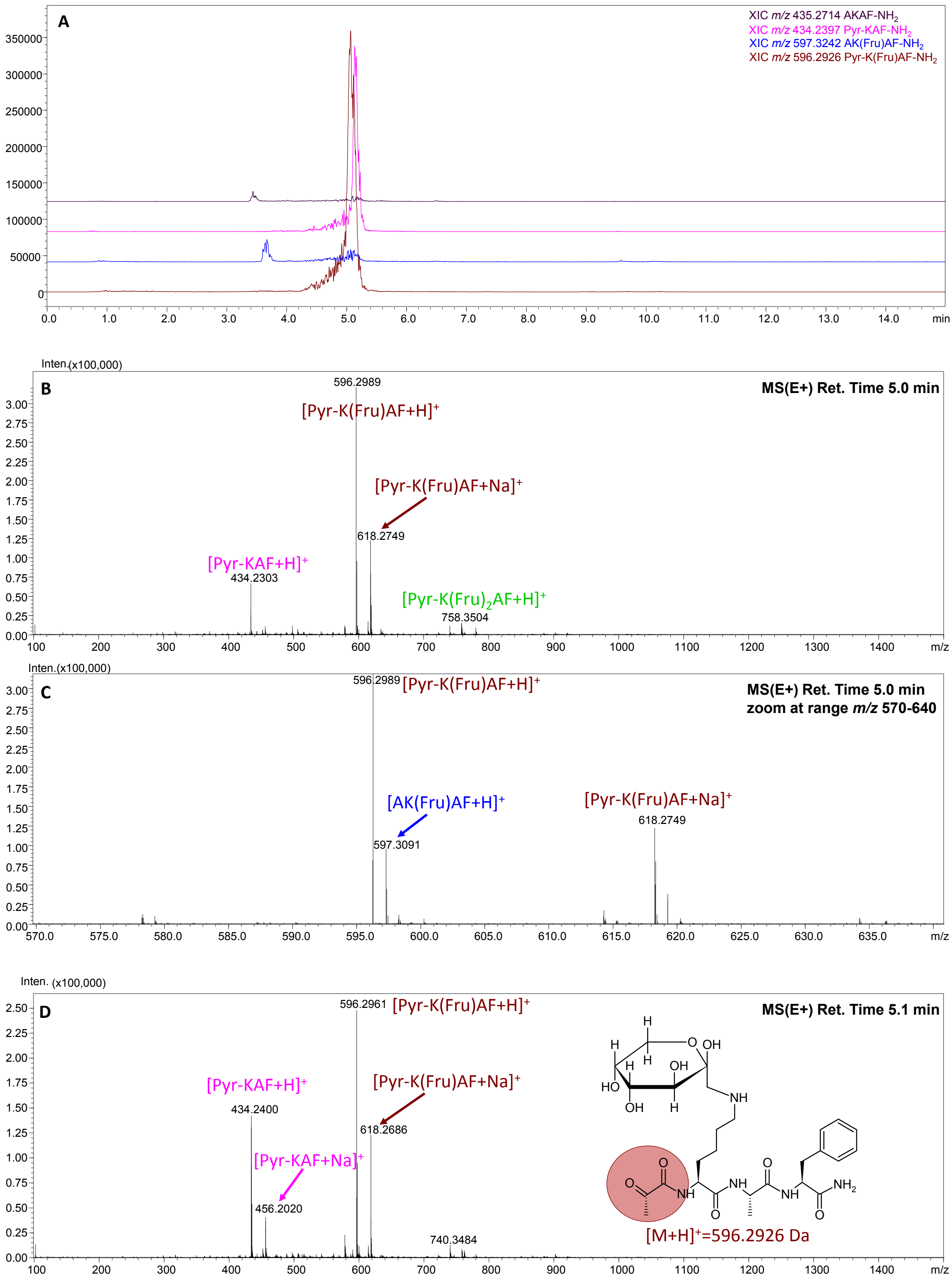

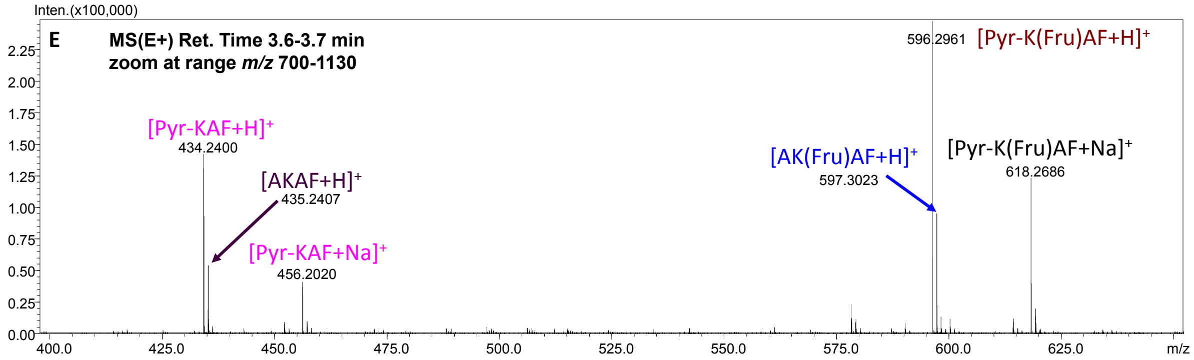

Fig. S20 LC-MS chromatogram of crude mixture of H-AKAF-NH_2 after reaction with glucose in conditions: 120°C in 4h (A); ESI-MS spectra for all identified signals on chromatogram (B-E).

H-AK(Fru)AF-NH$_2$: conditions: lactose, 120°C in 4h

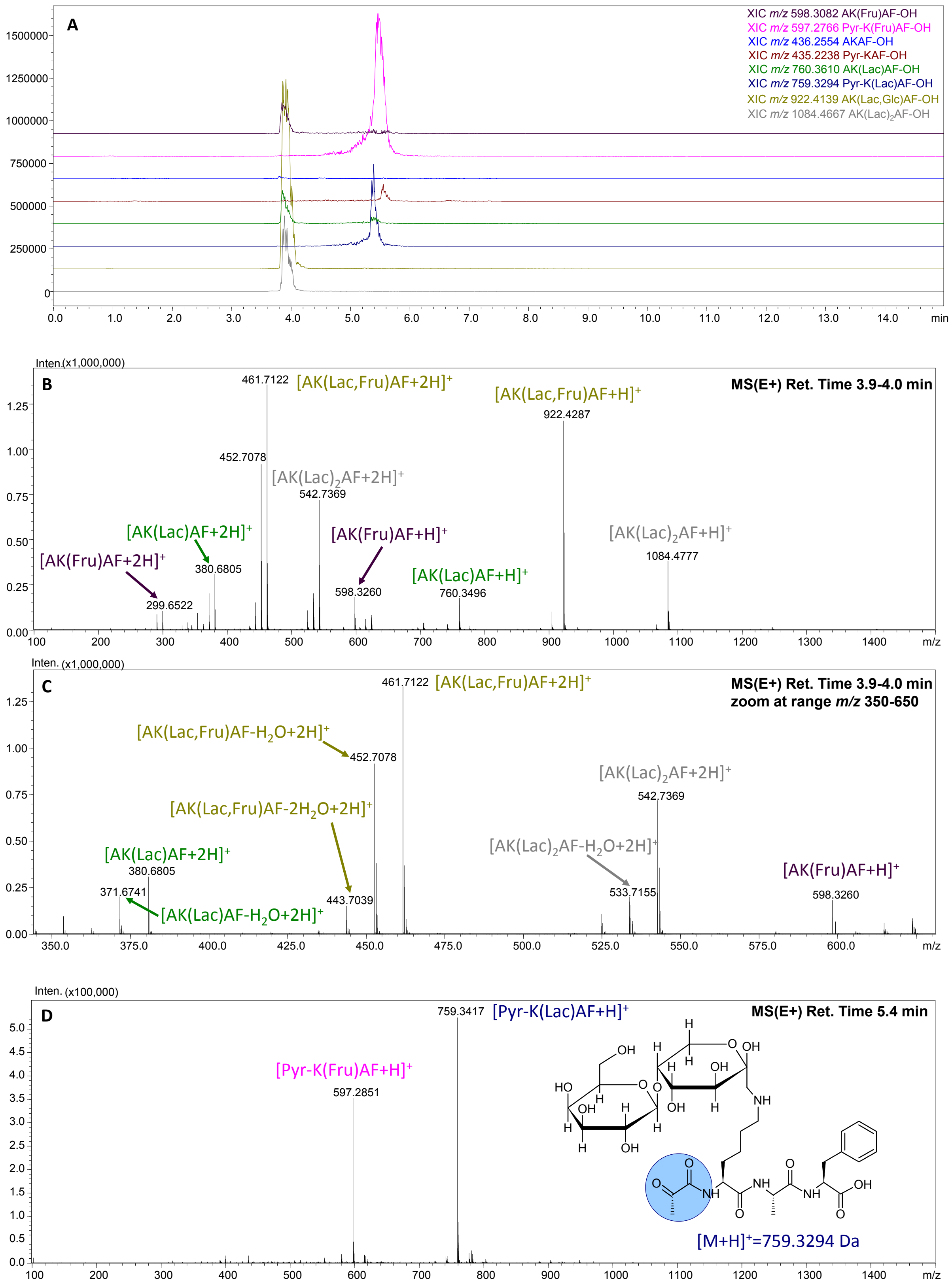

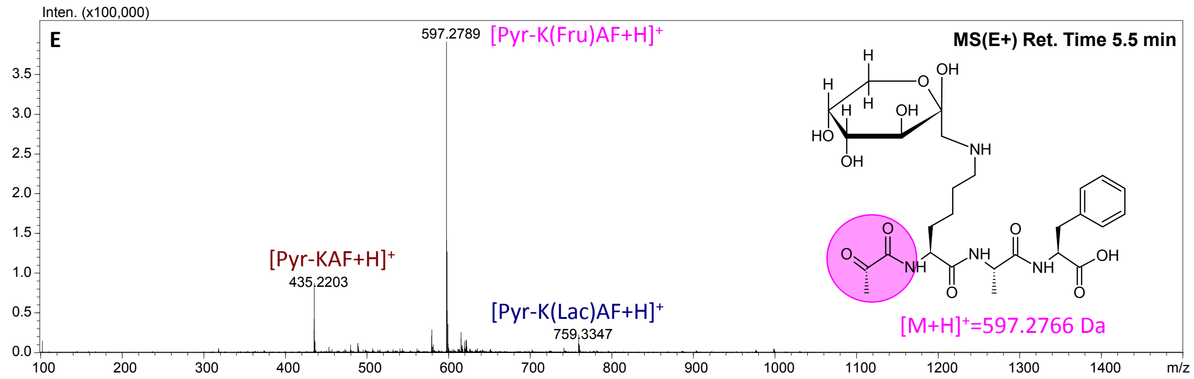

Fig. S21 LC-MS chromatogram of crude mixture of H-AK(Fru)AF-OH after reaction with lactose in conditions: 120°C in 4h (A); ESI-MS spectra for all identified signals on chromatogram (B-E).

Table 1.

Reaction conditions		Amadori products	Deaminated products
AKAF-NH_2	80°C, 20 min without sugar	✗	✗
	120°C, 4 h without sugar	✗	✗
AK(Fru)AF-OH	80°C, 20 min without sugar	✓	✗
	120°C, 4 h without sugar	✓	✗
AKAF-NH_2	80°C, 20 min with lactose	✗	✗
	120°C, 4 h with lactose	✓	✓
AKAF-NH_2	80°C, 20 min with glucose	✓	✗
	120°C, 4 h with glucose	✓	✓
AK(Fru)AF-OH	80°C, 20 min with lactose	✓	✗
	120°C, 4 h with lactose	✓	✓

✗ Absent in a sample ✓ Present in a sample
